# Structural insights into the human RyR2 N-terminal region involved in cardiac arrhythmias

**DOI:** 10.1107/S1399004714020343

**Published:** 2014-10-23

**Authors:** Ľubomír Borko, Vladena Bauerová-Hlinková, Eva Hostinová, Juraj Gašperík, Konrad Beck, F. Anthony Lai, Alexandra Zahradníková, Jozef Ševčík

**Affiliations:** a Department of Biochemistry and Structural Biology, Institute of Molecular Biology, Slovak Academy of Sciences, Dúbravská cesta 21, 845 51 Bratislava, Slovakia; b Cardiff University School of Dentistry, Heath Park, Cardiff CF14 4XY, Wales; c Department of Cardiology, Wales Heart Research Institute, Cardiff University School of Medicine, Cardiff CF14 4XN, Wales; d Department of Muscle Cell Research, Institute of Molecular Physiology and Genetics, Slovak Academy of Sciences, Vlárska 5, 833 34 Bratislava, Slovakia

**Keywords:** human ryanodine receptor 2, X-ray and SAXS structure, arrhythmogenic mutations, molecular modelling, phosphoprotein phosphatase I binding site

## Abstract

Human ryanodine receptor 2 (hRyR2) mediates calcium release from the sarcoplasmic reticulum, enabling cardio­myocyte contraction. The N-terminal region of hRyR2 (amino acids 1–606) is the target of >30 arrhythmogenic mutations and contains a binding site for phosphoprotein phosphatase 1. Here, the solution and crystal structures determined under near-physiological conditions, as well as a homology model of the hRyR2 N-terminal region, are presented. The N-terminus is held together by a unique network of interactions among its three domains, A, B and C, in which the central helix (amino acids 410–437) plays a prominent stabilizing role. Importantly, the anion-binding site reported for the mouse RyR2 N-terminal region is notably absent from the human RyR2. The structure concurs with the differential stability of arrhythmogenic mutations in the central helix (R420W, I419F and I419F/R420W) which are owing to disparities in the propensity of mutated residues to form energetically favourable or unfavourable contacts. In solution, the N-terminus adopts a globular shape with a prominent tail that is likely to involve residues 545–606, which are unresolved in the crystal structure. Docking the N-terminal domains into cryo-electron microscopy maps of the closed and open RyR1 conformations reveals C^α^ atom movements of up to 8 Å upon channel gating, and predicts the location of the leucine–isoleucine zipper segment and the interaction site for spinophilin and phosphoprotein phosphatase 1 on the RyR surface.

## Abstract 2

X-ray and solution structures of the human RyR2 N-terminal region were obtained under near-physiological conditions. The structure exhibits a unique network of interactions between its three domains, revealing an important stabilizing role of the central helix.

## 1. Introduction   

Ryanodine receptors (RyRs) are large homotetrameric calcium-permeant ion channels in the membrane of the endoplasmic/sarcoplasmic reticulum (ER/SR). The isoforms RyR1 and RyR2 are expressed primarily in skeletal and cardiac muscle, respectively, while the RyR3 isoform is more widely expressed (Sorrentino & Volpe, 1993 ▶). Cardiac RyR2 channels open in response to the action potential during systole and mediate excitation–contraction coupling by effecting massive release of calcium ions from the SR lumen into the cytosol of cardiac myocytes (Bers, 2002 ▶). During diastole, RyR2 activity is very low and produces a small diastolic calcium leak (Bers, 2002 ▶). Genetic diseases leading to cardiac arrhythmia [catecholaminergic polymorphic ventricular tachycardia (CPVT1) and arrhythmogenic right ventricular dysplasia (ARVD2)] are caused by RyR2 mutations that increase the diastolic calcium leak (Durham *et al.*, 2007 ▶; Yano *et al.*, 2006 ▶).

The RyR2 ion-conducting pore is located at the C-terminal end of the protein (Bhat *et al.*, 1997 ▶); however, >30 arrhythmo­genic mutations (http://www.fsm.it/cardmoc/) are located within the N-terminal region (amino acids 77–466; George *et al.*, 2007 ▶). Likewise, mutations in the RyR1 N-terminal region cause skeletal muscle diseases, malignant hyperthermia and central core disease (Durham *et al.*, 2007 ▶). A peptide corresponding to amino acids 163–195 of RyR2 (in which five disease-causing mutations are found) binds to RyRs and activates diastolic calcium release (Tateishi *et al.*, 2009 ▶). In RyR1, activation of calcium release by a peptide from the central region (amino acids 2442–2477) is suppressed by the N-terminal peptide DP3 (amino acids 324–351; Yamamoto *et al.*, 2000 ▶). Therefore, it has been proposed that in both RyR1 and RyR2 the N-terminal region allosterically regulates channel activity by interacting with the central part of the protein.

It is known that the RyR N-terminal region contains three domains named Ins145_P3_rec, MIR and RIH according to the Pfam database (Bauerová-Hlinková *et al.*, 2011 ▶). These domains are conserved in RyR channels and inositol trisphosphate receptor channels (IP3Rs). In the latter, the domains are responsible for binding of the agonist inositol trisphosphate. One of the prerequisites to understand the role of the N-terminal region in regulating the activity of RyRs is solution of its structure at the atomic level. In contrast to those of the IP3Rs (Lin *et al.*, 2011 ▶) and the skeletal RyR1 isoform (Tung *et al.*, 2010 ▶), the structure of this region of RyR2 was not available until recently (Kimlicka, Tung *et al.*, 2013 ▶). The slow progress in the purification and crystallization of the RyR2 N-terminal region has been attributed to the presence of a chloride-binding site in the cardiac RyR2 that required the presence of supraphysiological chloride concentrations for protein solubility (Kimlicka, Tung *et al.*, 2013 ▶).

Here, we present the X-ray and SAXS structures of the human RyR2 N-terminal fragment encompassing amino acids 1–606 (hRyR2^1–606^) obtained at physiological chloride levels. No Cl^−^ anion was found in hRyR2^1–606^ and the structure did not show any evidence of a chloride-binding site as had been observed in the mouse RyR2^1–547^ structure (Kimlicka, Tung *et al.*, 2013 ▶). Pronounced differences between hRyR2^1–606^ and the chloride-containing mRyR2^1–547^ were found at their A/B, B/C and A/C interfaces, notably in the region of the proposed chloride-binding site (Kimlicka, Tung *et al.*, 2013 ▶). The hRyR2^1–606^ structure was docked into the cryo-electron microscopy (cryo-EM) map of the native RyR1 to shed light on its interactions with other domains and with the associated regulatory protein phosphoprotein phosphatase 1 (PP1). To understand the role of Ile419 and Arg420 located in the central helix (amino acids 410–437) we used circular-dichroism (CD) spectroscopy, thermal shift assay and analytical gel filtration to characterize their mutations I419F, R420W and I419F/R420W.

## 2. Materials and methods   

### 2.1. Crystal structure   

Preparation of hRyR2^1–606^ protein, crystallization, data collection and processing have been described in detail in Borko *et al.* (2013 ▶). Substitution mutants were prepared using the QuikChange protocol (Stratagene). Crystals formed in hanging drops from a solution consisting of 20 m*M* Tris–HCl pH 7.5, 150 m*M* NaCl, 10% glycerol, 7 m*M* β-mercapto­ethanol, 1 m*M* CHAPS, 0.1% betaine mixed with reservoir solution (100 m*M* HEPES pH 6.9, 200 m*M* ammonium formate, 21% PEG 3350) in a 2:1 ratio.

Data were collected to 2.39 Å resolution at 100 K at the BESSY II synchrotron-radiation source, Berlin, Germany using 15% ethylene glycol as cryoprotectant. Data collection was performed using a MAR345dtb image plate. Data were processed with *iMosflm* (Powell *et al.*, 2013 ▶) and were scaled by *SCALA* (Evans, 2011 ▶). The crystals belonged to space group *P*4_2_2_1_2, with unit-cell parameters *a* = 75.45, *b* = 75.45, *c* = 248.84 Å, α = β = γ = 90° and one molecule in the asymmetric unit (Borko *et al.*, 2013 ▶). The structure was solved by molecular replacement with *MOLREP* (Vagin & Teplyakov, 2010 ▶) using oRyR1^1–559^ (PDB entry 2xoa; Tung *et al.*, 2010 ▶) as a search model. The structure was built using *Buccaneer* (Emsley & Cowtan, 2004 ▶) and refined with *REFMAC* 5.6.0117 (Murshudov *et al.*, 2011 ▶) including TLS refinement (Winn *et al.*, 2001 ▶). Three TLS groups (10–223, 224–408 and 409–544) were used. Refinement of the structure was altered by inspecting the map, correcting the amino-acid sequence and manually building the parts that were not built by *Buccaneer*. Water molecules were automatically built by *Coot* (Emsley & Cowtan, 2004 ▶) into electron densities at a ≥1σ level that were at hydrogen-bonding distances from protein atoms or other water molecules. Data-collection and refinement statistics of the hRyR2^1–606^ structure are summarized in Table 1 ▶.

### 2.2. Quality of the final protein model   

**Figure 1:**
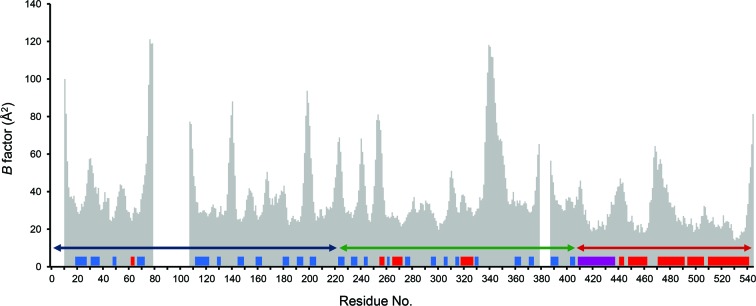
Temperature factors of C^α^ atoms as a function of residue number. The secondary-structure elements are shown as rectangles (blue, β-sheet; red, α-helix). The central α-helix is shown in purple. The gaps represent residues that are missing in the structure. Domains are indicated by double arrows (A, blue; B, green; C, red).

Crystal structure refinement (Table 1 ▶) converged with *R* and *R*_free_ of 22.4 and 26.1%, respectively. The refined model had good geometry with r.m.s.d.s from ideal bond lengths and angles of 0.008 Å and 1.180°, respectively. The estimated overall coordinate error based on maximum likelihood (ESU) was 0.205 Å. The Ramachandran diagram (*RAMPAGE*; Lovell *et al.*, 2003 ▶) showed that 99.2% of residues were in the allowed regions (Table 1 ▶). The average *B* factor for main-chain atoms and for all atoms was 36.9 and 37.7 Å^2^, respectively. The Wilson plot *B* factor was 50.4 Å^2^. The bar diagram (Fig. 1 ▶) shows the temperature factors for C^α^ atoms as a function of residue number. The average *B* factor for main-chain atoms and for all atoms was 36.9 and 37.9 Å^2^, respectively. The Wilson plot *B* factor was 50.4 Å^2^.

### 2.3. Small-angle X-ray scattering structure   

The sample was prepared according to Borko *et al.* (2013 ▶). The sample quality was tested by dynamic light scattering (DLS; Zetasizer Nano-S) and size-exclusion chromatography (SEC; GE ÄKTA FPLC, Superose 12 HR10/30) at 5–10°C. Prior to SAXS data collection the protein sample was diluted at ratios of 1:1, 1:2, 1:3, 1:4 and 1:5 with the sample buffer without detergents. The protein concentration during the measurement was in the range 2–10 mg ml^−1^. SAXS data were collected on beamline X33, DESY, Hamburg (see Supporting Information^1^ for beamline characteristics). The SAXS data were processed with programs from the *ATSAS* 2.5 package (Supplementary Fig. S1). Initial processing and analysis were performed with *PRIMUS* (Konarev *et al.*, 2003 ▶). The radius of gyration (*R*_g_) was estimated by Guinier approximation and from the pair-distribution function *P*(*r*). Information about the degree of protein disorder was obtained from the Kratky plot. The particle volume was calculated using the Porod invariant and the molecular weight was estimated from the Porod volume. *Ab initio* modelling was performed with *GASBOR* (Svergun *et al.*, 2001 ▶) using ten repetitions with one scattering curve, with χ^2^ between 0.9 and 1. The normalized spatial discrepancy (NSD) of all models was compared using *DAMSEL* (Volkov & Svergun, 2003 ▶). The average NSD was 1.50 ± 0.07.

### 2.4. Biophysical analysis   

A thermal shift assay was performed using an iCycler IQ5 PCR Thermal Cycler (Bio-Rad) operated with the *IQ*5 software v.2.1 using a 1:1000 dilution of the 5000× SYPRO Orange (Invitrogen) protein dye solution and 5 µg protein in 25 µl buffer. The initial and final holding steps were set to 10 s at 20 and 80°C, respectively; the ramp increment was set to 1°C and the scanning time to 10 s. CD spectra were collected at 4–40°C at a protein concentration of ≃15 µ*M* using an Aviv Model 215 instrument (Aviv Biomedical Inc., Lakewood, New Jersey, USA) with a 0.01 or 0.02 cm path-length cell. Detailed information is in the Supporting Information. Secondary-structure content was estimated using the *CDSSTR* (Johnson, 1999 ▶) algorithm as implemented on *DichroWeb* (Whitmore & Wallace, 2008 ▶) using the SP175 reference spectra (Lees *et al.*, 2006 ▶).

### 2.5. Modelling the missing residues   

Homology models of hRyR2^1–606^ that contained all residues, including those missing from the X-ray structure, were built with the *I-TASSER* protein structure and function prediction server (http://zhanglab.ccmb.med.umich.edu/I-TASSER/; Roy *et al.*, 2010 ▶). The confidence score (C-­score), based on the quality of threading alignments and the convergence of structural assembly refinement simulations, was used for estimating the accuracy of structure predictions. The crystal structures of the N-terminal three domains of skeletal muscle RyR1 (PDB entry 2xoa; Tung *et al.*, 2010 ▶) and the ligand-binding domain of an InsP3 receptor (PDB entry 3t8s; Lin *et al.*, 2011 ▶) were used as templates. Of the five resulting models, the one with an extended C-terminus imitating the shape of the SAXS envelope (C-score of −2.88) was used for docking into the envelope. The *I-TASSER* model with the best C-score of −0.10 was used for completing the final model (PDB entry 4jkq) with the residues missing in the X-ray structure. The resulting model was minimized in *Chimera* (Pettersen *et al.*, 2004 ▶) and used for docking into the RyR1 cryo-EM maps. Figures were prepared using *Chimera* unless indicated otherwise.

### 2.6. Docking the hRyR2^1–606^ structure into SAXS and cryo-EM electron-density maps   

The SAXS structure was transformed into an electron-density map with a resolution of 9.38 Å using *pdb*2*vol* from the *SITUS* 2.7 package (Wriggers, 2010 ▶). The hRyR2^1–606^ crystal structure was docked into the SAXS envelope using *colores* (*SITUS 2.7*). The hRyR2^1–606^ homology model with the C-terminal tail (amino acids 519–606) was docked manually into the SAXS envelope. Cross-correlation was maximized by *collage* (*SITUS* 2.7). The shapes of the predicted atomic structure of hRyR2^1–606^ and the SAXS envelope were compared by *CRYSOL* from the *ATSAS* 2.5 package, giving a χ^2^ of 2.72. Docking of the hRyR2^1–606^ crystal structure, its individual domains and the homology model into cryo-EM maps of RyR1 was performed by *colores* using Laplace filtering.

## 3. Results   

### 3.1. Overview of the hRyR2^1-606^ crystal structure   

**Figure 2:**
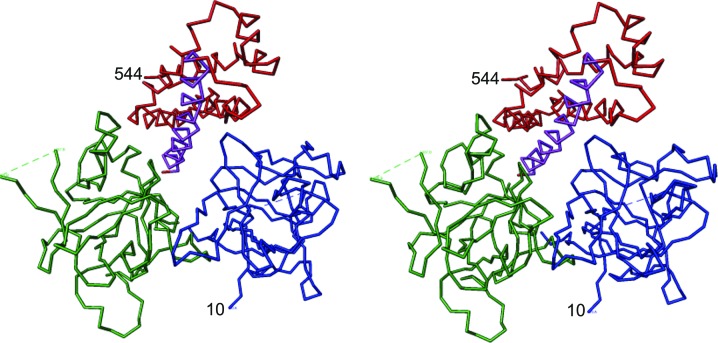
A stereoview of the 4jkq structure. The figure shows the C^α^ trace of the hRyR2^1–606^ structure consisting of the three domains A (blue), B (green) and C (red) and the central helix (amino acids 410–437; purple).

**Figure 3:**
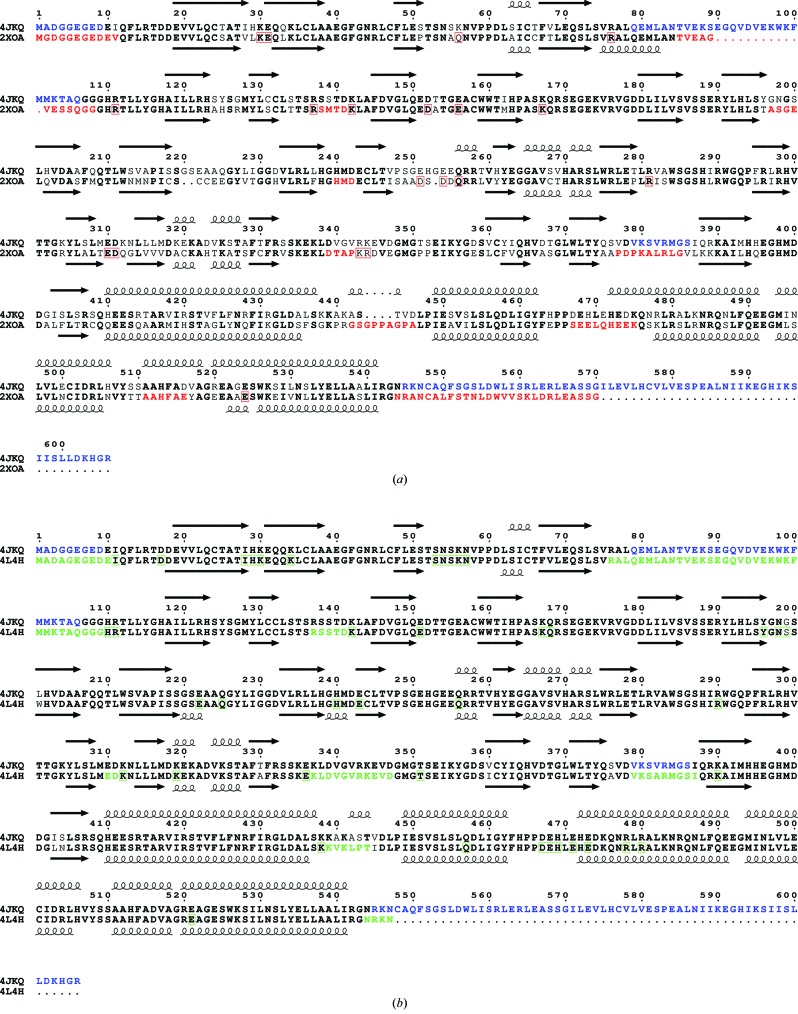
Alignment of N-terminal RyR regions. (*a*) hRyR2^1–606^ and oRyR1^1–559^, (*b*) hRyR2^1–606^ and mRyR2^1–547^. Identical amino acids are in bold. Secondary-structure elements determined from the X-ray structures are shown as arrows (β-strands) and helices. Blue, green and red colours indicate the residues that are missing in 4jkq, 4l4h and 2xoa, respectively. Missing side chains are boxed.

The structure of hRyR2^1–606^ determined at 2.39 Å consists of three domains: A (residues 10–223) and B (224–408) adopting a β-trefoil fold, and C (409–544) formed by a bundle of five α-helices (Fig. 2 ▶). Domains A and B correspond to the Pfam domains Ins145_P3_rec and MIR, and domain C includes the RIH domain. The first α-helix of domain C (referred to as the central helix) is positioned in the centre of the structure and forms contacts with all three domains. This helix is not part of the RIH domain (amino acids 451–655; Bauerová-Hlinková *et al.*, 2011 ▶). The alignment between N-terminal sequences, secondary-structure elements and unresolved residues of the present hRyR2 structure (PDB entry 4jkq; amino acids 1–606), with the homologous structures of rabbit RyR1 (2xoa; amino acids 1–559; Tung *et al.*, 2010 ▶) and mouse RyR2 (4l4h; amino acids 1–547; Kimlicka, Tung *et al.*, 2013 ▶) is shown in Figs. 3 ▶(*a*) and 3 ▶(*b*). Residues 1–9, 79–106, 379–386 and 545–606 are not visible in the hRyR2^1–606^ structure. The high *B*-factor values for C^α^ atoms (Fig. 1 ▶) represent segments of the structure which are likely to be highly flexible owing to the absence of their binding partners present in the native hRyR2. High flexibility is also responsible for the gaps in the structure where electron density was not interpretable.

The hRyR2^1–606^ molecule contains ten cysteine residues, of which the two pairs Cys36/Cys65 and Cys131/Cys158 are well positioned to form disulfide bonds; no S–S bridge could be seen in the structure, which might be owing to the presence of β-mercaptoethanol (7 m*M*), which was used during purification and crystallization.

Differences between the N-terminal structures of hRyR2 and those of mRyR2 and oRyR1 are analysed in detail in Table 2 ▶. Here, the deviations between C^α^ atoms of the three analysed structures superimposed either as a whole or by their individual domains are shown. The rows in the table show the differences in the relative position of a given region of the three compared structures, characterized by the r.m.s.d. of their C^α^ atoms after the superposition of individual substructures. As expected, the r.m.s.d.s are the smallest when the same regions are both superimposed and compared (bold numbers), and show that while domains A and C are structurally very similar in all three structures, there are large differences between the structures of domain B. The largest difference is in the region of the long loop composed of residues 333–361 in domain B, which is only partially resolved in 4l4h and 2xoa. Columns in the table show the effect of superimposing a given region on the r.m.s.d. of the other regions. Superposition of the A domains of 4l4h and 2xoa onto 4jkq has the largest effect on the relative position of the remaining regions. It leads to a significant increase of the r.m.s.d. between the other corresponding domains by up to 3 Å and results in rotation of the structures 4l4h and 2xoa by 2.5–6.7° relative to the structure 4jkq. A similar shift of the relative position of domains A and B is observed when domains C are superimposed. The effect of superimposing domain B is less prominent. Superposition of the structures based on domain A is also illustrated in Supplementary Fig. S2.

Upon docking the hRyR2^1–606^ structure into RyR cryo-EM maps (see Supporting Information), four symmetrical copies of the structure form the central rim of the RyR (Supplementary Movie S1), as previously reported for 2xoa (Tung *et al.*, 2010 ▶). Individual faces of the structure are shown relative to their positions in the whole RyR molecule, with the top and bottom surfaces pointing to the cytoplasm and to the SR membrane, respectively.

### 3.2. Electrostatic potential distribution   

Despite a high sequence identity and similarity of the X-ray structures of hRyR2^1–606^, mRyR2^1–547^ and oRyR1^1–559^, their overall net charge calculated *in vacuo* differs and reaches values of −9, −13.9 and −5, respectively. This discrepancy is caused by the absence of 33 residues and 41 side chains in the mRyR2^1–547^ structure and approximately the same number of residues and 19 side chains in oRyR1^1–559^ located predominantly on the surface, which are present in the hRyR2^1–606^ structure. Thus, the calculated charges of the mRyR2^1–547^ and oRyR1^1–559^ structures substantially differ from the true value.

**Figure 4:**
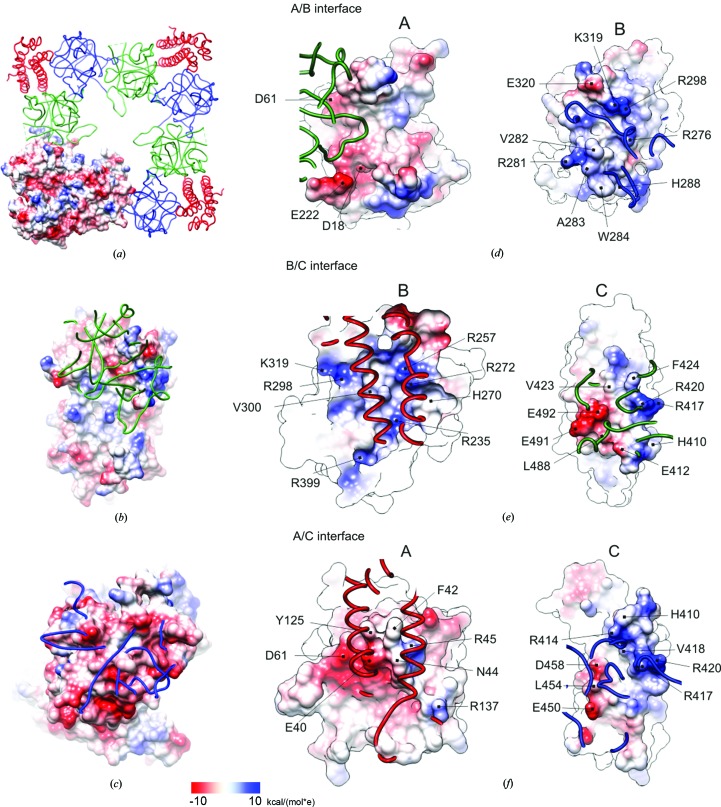
Electrostatic potential distribution of hRyR2^1–606^ surface. (*a*) A view of the four monomers of hRyR2^1–606^ that form the central rim of the RyR, with one of the monomers showing the potential distribution. Domains A, B and C are shown in blue, green and red, respectively. (*b*) A view of the potential distribution (blue, positive; red, negative) of the surface of domain A that interacts with domain B of the neighbouring hRyR2^1–606^ monomer (green ribbon). (*c*) A view of the potential distribution of the surface of domain B that interacts with domain A of the neighbouring hRyR2^1–606^ monomer (blue ribbon). (*d*–*f*) Electrostatic potential distribution at the interfaces A/B (*d*), B/C (*e*) and A/C (*f*) of hRyR2^1–606^. One side of the interface is shown as a electrostatic potential surface, while the complementary side is shown as a ribbon (blue, green and red for domains A, B and C, respectively). The two views were created by a 180° rotation around the vertical axis. Residues with a substantial electrostatic potential are indicated. Most of the interactions between domains A/C and B/C involve residues from the central helix (Table 3 ▶).

We have analysed the electrostatic potential distribution of hRyR2^1–606^ homotetramer interfaces (Figs. 4 ▶ *a*, 4 ▶ *b* and 4 ▶ *c*). Figs. 4 ▶(*b*) and 4 ▶(*c*) show a detailed view of the surfaces of domains A (Fig. 4 ▶ *b*) and B (Fig. 4 ▶ *c*) that participate in the intermonomer interaction. The interacting surfaces of domains A and B show a prevalence of positive and negative potentials, respectively, suggesting a significant role of electrostatic interactions in RyR2 central rim tetramerization.

### 3.3. Interdomain interfaces and the putative chloride-binding site   

**Figure 5:**
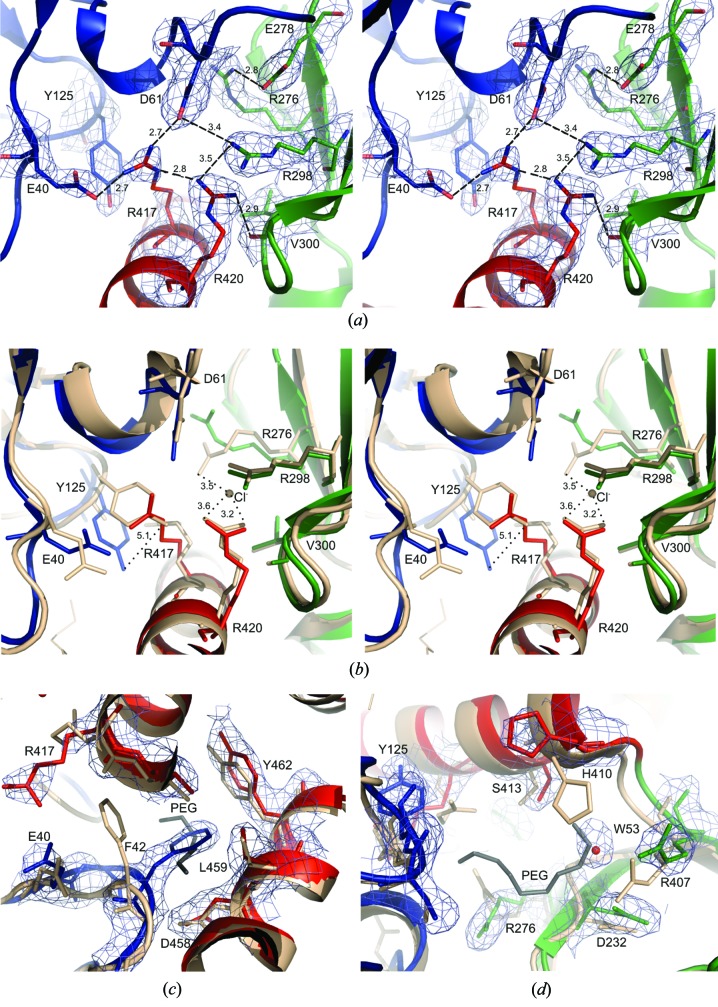
Conformation of residues at the putative binding sites of RyR2. (*a*) A stereoview of the salt-bridge/hydrogen-bond network of hRyR2^1–606^ stabilizing the A/B/C interface. Hydrogen bonds are shown as dashed lines. (*b*) A stereoview of the superposition of the chloride-binding site of mRyR2^1–547^ (light beige) with the equivalent site of hRyR2^1–606^. There are major differences in the conformations of the Glu40, Tyr125, Arg276 and Arg417 side chains; the mRyR2^1–547^ chloride anion (indicated) has no equivalent in the hRyR2^1–606^ structure. (*c*, *d*) Comparison of the hRyR2^1–606^ structure close to the A/C interface (*c*) and the A/B/C interface (*d*) with the putative PEG binding sites of the mRyR2^1–547^ structure. In the 4jqk structure, the Phe42 side chain is located in one of the PEG binding sites found in mRyR2^1–547^ (*c*). In the region of the other putative PEG binding site, no electron density is present in 4jkq except for a single water molecule (*d*). Residues from domains A, B and C of hRyR2^1–606^ are shown in blue, green and red, respectively. Residues of mRyR2^1–547^ are shown in light beige. PEG is coloured in grey. Electron density is drawn at the 1σ level. This figure was prepared with *PyMOL* (Schrödinger).

Analysis of the interfaces between domains A/B, A/C and B/C is shown in Table 3 ▶. Most of the interactions between domains A/C and B/C involve the central helix, strongly suggesting a significant role of this helix in maintaining the integrity of the N-terminal fragment. For all three interdomain interfaces in hRyR2^1–606^, the juxtaposed protein surfaces have complementary electrostatic potentials (Figs. 4 ▶ *d*, 4 ▶ *e* and 4 ▶ *f*), suggesting an important role of polar and charged interactions. The interdomain A/B/C interactions in hRyR2^1–606^ notably differ from those in mRyR2^1–547^ owing to the presence of a Cl^−^ anion in the latter (Figs. 5 ▶ *a* and 5 ▶ *b*). As a result, the side chains of Glu40, Tyr125, Arg276 and Arg417 in hRyR2^1–606^ adopt different conformations from those in mRyR2^1–547^. Thus, the N atoms of Arg417 are within hydrogen-bonding distances of carboxyl O atoms of the domain A aspartate, glutamate and serine (Asp61 OD2, Glu40 OE2 and Ser63 OG). The Arg420 NH1 atom forms a hydrogen bond to the main-chain O atom of Val300 and Arg298 NH1 of domain B. Moreover, the guanidine group of Arg420 makes a face-to-face stacking interaction with that of Arg298. All of the above-mentioned contacts indicate the importance of Arg417 and Arg420 for maintaining a stable A/B/C domain arrangement in the hRyR2^1–606^ structure (Figs. 5 ▶ *a* and 5 ▶ *b*). The importance of the central helix is apparent also from its relatively low *B* factors (Fig. 1 ▶).

The electrostatic interactions between domains A, B and C observed in the present structure are replaced in the mRyR2^1–547^ structure (Kimlicka, Tung *et al.*, 2013 ▶) by contacts of Arg420, Arg298 and Arg276 with a Cl^−^ anion, for which the authors proposed a crucial role in mediating interdomain interactions and the stability of the whole structure. Significantly, despite the presence of chloride anions in our crystallization solution (Borko *et al.*, 2013 ▶), there was no electron density in the close proximity of Arg420 in the hRyR2^1–606^ structure to be assigned to a Cl^−^ anion. In contrast to mRyR2^1–547^, the Arg276 guanidine in hRyR2^1–606^ forms a hydrogen bond to Glu278 OE2. Furthermore, the OH atoms of Tyr125 in superimposed hRyR2^1–606^ and mRyR2^1–547^ are almost 5 Å apart. In the hRyR2^1–606^ structure the position of the Tyr125 side chain of mRyR2^1–547^ is occupied by Arg417 (Fig. 5 ▶ *b*).

In oRyR1 (Tung *et al.*, 2010 ▶), the equivalent of Arg420 in hRyR2 is His405, for which the side chain, in contrast to Arg420, forms neither polar nor charged contacts with the surrounding residue. While in oRyR1 the hub of the salt-bridge/hydrogen-bond network stabilizing the A/B/C interface is formed by Asp61 from the A domain, in hRyR2 the hub is formed by the tandem of Arg417 and Arg420 from the central helix (Fig. 5 ▶ *a*). Thus, the interdomain interface interactions of hRyR2 substantially differ from other N-terminal RyR structures known so far.

### 3.4. Other putative binding sites   

Two putative binding sites situated close to the A/C and A/B/C interfaces have been reported for mRyR2^1–547^; the electron density in this region was assigned to PEG molecules (Kimlicka, Tung *et al.*, 2013 ▶). Despite the use of a rather high PEG 3350 concentration in our crystallization solution (Borko *et al.*, 2013 ▶), no PEG molecules were identified in the hRyR2^1–606^ structure. In one of the PEG-binding sites proposed for mRyR2^1–547^, we find that the side chain of Phe42 is located at this position in the 4jqk structure (Fig. 5 ▶ *c*). In the region of the other putative PEG-binding site, no electron density is present in 4jkq except for a single water molecule (Fig. 5 ▶ *d*).

### 3.5. Mutations associated with ARVD2 and CPVT1   

The new structural details revealed for hRyR2^1–606^ enabled assessment of the potential role of several residues, mutation of which result in CPVT/ARVD2 (http://www.fsm.it/cardmoc/). Based on the analysis of the structure, we could address the likely effect of mutations L62F, P164S, R169Q, V186M, I217V, E243K, F329L, R332W, D400H, R414C/L, T415R, I419F, R420W/Q and L433P; Supplementary Table S1). Of all the known residues undergoing arrhythmogenic mutations in this region, there is only one, Met81 located in the dynamic α-helix (Amador *et al.*, 2013 ▶), that was not present in the hRyR2^1–606^ structure.

**Figure 6:**
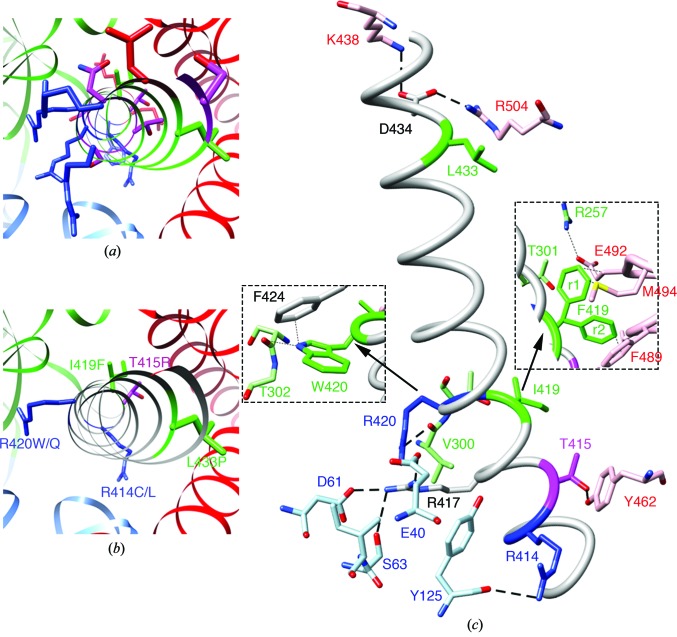
The central helix and its mutations. (*a*) Position of the central helix amidst the A (blue), B (green) and C (red) domains. Conformation of the most important amino acids is shown. The ribbon of the central helix is colour-coded by the properties of the respective amino acids: blue, positively charged; red, negatively charged; purple, polar; green, hydrophobic. (*b*) Central helix residues susceptible to mutations associated with cardiac arrhythmias in the hRyR2^1–606^ structure. (*c*) The important amino acids forming a charge network stabilizing the structure. Right inset: the two most probable conformations of Phe419 in the I419F mutant (rotamers r1 and r2) show clashes with neighbouring residues, explaining the thermal stability decrease of the structure. Left inset: Arg420 points to solution; this may also occur for R420W in the mutant, explaining why this mutation has not affected the thermal stability. The closest contacts of Trp420 in the R420W mutant are with the Phe424 side chain (≃2.5 Å) and the Thr302 main chain (≃3 Å). Central helix amino-acid residues undergoing mutations are coloured as follows: blue, positively charged; red, negatively charged; purple, polar; green, hydrophobic.

In all reported structures of the RyR N-terminal region encompassing the first ∼600 amino acids, the central α-helix is prominent. This helix, positioned between the ABC domains, forms the beginning of domain C and is not a part of any Pfam-predicted domain (Bauerová-Hlinková *et al.*, 2011 ▶). The interface of the central helix with the remainder of domain C is composed of hydrophobic and negatively charged side chains, while the part facing domains A and B is composed mostly of hydrophobic and positively charged side chains (Fig. 6 ▶ *a*). The central helix interacts with the surrounding domains through polar, charged and hydrophobic interactions (Table 4 ▶). In hRyR2, five amino acids in the central α-helix are subject to a total of seven disease-associated mutations (Fig. 6 ▶ *b* and Table 4 ▶). These amino acids are actively involved in the network stabilizing the N-terminus structure (Fig. 6 ▶ *c*). Ile419, which is fully conserved in RyR isoforms from different mammalian species and exhibits pathogenic mutations in both RyR1 and RyR2, forms contacts with residues of domain C. Introducing Phe in position of I419 likely causes severe steric clashes resulting in destabilization of the structure (Fig. 6 ▶ *c*, right inset). The most probable rotamer of Trp in position of Arg420 should adopt a conformation forming acceptable contacts with neighbouring residues, thus avoiding harmful structural changes (Fig. 6 ▶ *c*, left inset). Overall, in the central helix and amino acids that directly precede it (amino acids 400–409), there is a significantly higher frequency of arrhythmogenic mutations than in the remainder of the N-terminal fragment, emphasizing the importance of central helix integrity for proper RyR2 function.

**Figure 7:**
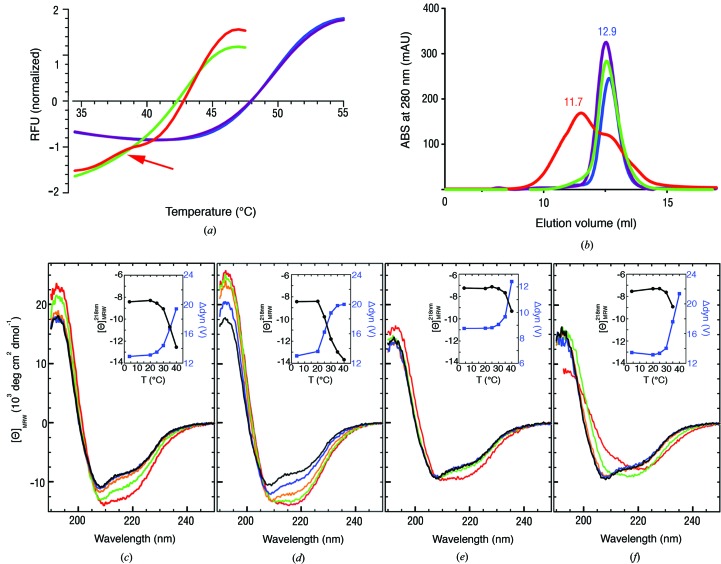
Stability of hRyR2^1–606^ and its mutants. (*a*) Normalized thermal stability curves of hRyR2^1–606^ (blue) and its mutants I419F (red), R420W (violet) and I419F/R420W (green), measured from 20 to 90°C. The transition around 38°C (indicated by the arrow) corresponds to the melting of Ile419 dimers. (*b*) Analytical SEC profile of hRyR2^1–606^ wild type (blue) and of I419F (red), R420W (purple) and I419F/R420W (green) mutants. The elution maximum at 11.7 ml corresponds to dimers of I419F. Elution maxima at 12.9 ml correspond to the wild type and mutant monomers. (*c*)–(*f*) Far-UV CD spectra of hRyR2^1–606^ wild type (*c*) and I419F (*d*), R420W (*e*) and I419F/R420W (*f*) mutants recorded at 4°C (black), 25°C (blue), 30°C (orange), 35°C (green) and 40°C (red). Insets show the ellipticities [Θ] (black) and the dynode voltage difference from the buffer baseline Δdyn (blue) at 218 nm as a function of the temperature.

We have successfully purified the cardiac disease-associated mutants of hRyR2^1–606^, I419F and R420W, and the double-mutant I419F/R420W. Thermofluor analysis of I419F and I419F/R420W (Fig. 7 ▶ *a*) showed a decrease in thermal stability by ∼6 and 8°C, respectively, compared with the wild type. The R420W mutation by itself did not induce a significant change of thermal stability (Fig. 7 ▶ *a*). Size-exclusion chromatography at 4°C showed a propensity of the I419F mutant to dimerize (a dimer:monomer ratio of 0:100% and 65:35% for the wild type and the I419F mutant, respectively; Fig. 7 ▶ *b*).

CD spectra of hRyR2^1–606^ and the mutant proteins I419F, R420W and I419F/R420W recorded at 4°C are shown in Figs. 7 ▶(*c*)–7(*f*). The spectrum of the I419F mutant was virtually identical to that of the wild-type protein, whereas the R420W and I419F/R420W mutant proteins exhibited slightly lower amplitudes. Deconvolution of the spectra resulted in an α-helical and β-strand content of 22 and 29%, respectively, for wild-type and I419F proteins, whereas the helical content was estimated to be ∼3% lower for the R420W and I419W/R420W mutants. The CD-based estimate of secondary structure for the wild-type protein is in excellent agreement with that of the crystal structure, which contains 21.8% α-helix and 29.5% β-strands.

CD spectra recorded at increasing temperatures showed anomalous behaviour: whereas protein unfolding is usually indicated by a decrease in the absolute amplitude, hRyR2^1–606^ and its variants showed the opposite effect (Figs. 7*c*–7*f* ▶). The increase in the CD amplitude was accompanied by an increase in the photomultiplier dynode voltage (insets in Figs. 7 ▶ *c*–7*f*), indicative of light scattering owing to the formation of aggregates. Whereas the wild­type and the double mutant protein exhibited aggregation at *T* > 30°C, the I419F and R420W mutants aggregated at *T* > 20°C and *T* > 35°C, respectively. Besides the central helix mutants we also analysed P164S and R176Q mutant proteins. Their CD spectra have shown no deviation from those of wild-type hRyR2^1–606^.

Docking of the X-ray structure and a homology-based model into the RyR1 cryo-EM map (Supplementary Fig. S3 and Supplementary Movie S1) indicates that domain C cannot be involved in interaction between hRyR2^1–606^ monomers *in situ*; thus dimerization can be viewed as a surrogate for interaction with another protein sequence outside of the 1–606 region.

### 3.6. Docking of the crystal structure and its model into the solution structure   

**Figure 8:**
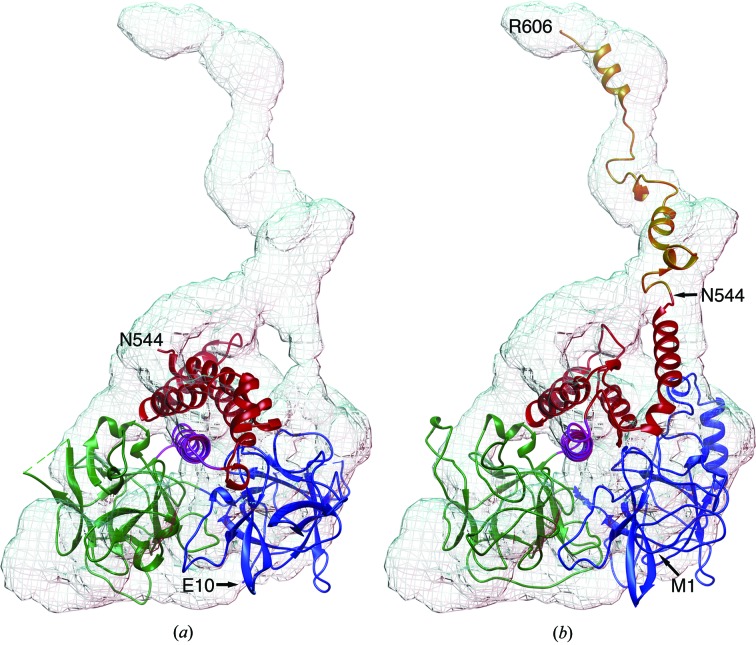
Solution structure of hRyR2^1–606^. Crystal structure of hRyR2^1–606^ (*a*) and the *I-TASSER* prediction model (*b*) fitted into the SAXS envelope. Blue, domain A, INS145_P3_rec; green, domain B, MIR; purple, central helix; red (amino acids 451–544) and orange (amino acids 545–606), parts of the RIH domain.

To resolve the topology of the molecule in solution, the structure of hRyR2^1–606^ was determined by SAXS. A globular topology was expected; however, the structure was a combination of a globule and a tail (Fig. 8 ▶). Docking of the crystal structure into the globular part of the SAXS envelope (Fig. 8 ▶ *a*) yielded four best orientations with cross-correlation coefficients of 0.60–0.64. In the optimal orientation (*R* = 0.64), the C-terminus of hRyR2^1–606^ (Gln544) was positioned next to the tail of the SAXS structure, suggesting that the residues unresolved in the crystal structure (amino acids 545–606) are located within the tail.

According to the *I-TASSER* prediction server, the RIH domain (amino acids 451–655) is formed by an armadillo motif composed of α-helices. Therefore, it is conceivable that the incomplete RIH domain is flexible, possibly leading to its partial unfolding. To verify this possibility, a set of five homology models of the complete hRyR^1–606^ sequence was constructed using the *I-TASSER* server. One model (C-score of −2.88) had an extended tail (amino acids 542–606). The model provided a cross-correlation coefficient of 0.65 after docking into the SAXS envelope (Fig. 8 ▶ *b*) and gave an acceptable *CRYSOL* fit (Supplementary Fig. S1) to the experimental SAXS data. Thus, it is highly likely that the tail observed in the SAXS structure is formed by the 61 C-terminal residues (amino acids 545–606) that are unresolved in the crystal structure.

### 3.7. Docking the crystal structure and its model into cryo-EM maps   

Docking the hRyR2^1–606^ structure into cryo-EM maps of the closed and open RyR1 suggests that this region undergoes substantial movement upon channel opening (Supplementary Fig. S3 and Supplementary Movie S1). The distances between nearby C^α^ atoms of neighbouring hRyR2^1–606^ monomers in the open conformation were on average larger by ≃3 Å than those in the closed conformation (Supplementary Table S2). Different distances between atom pairs in the closed and open conformation suggest that the central rim not only rotates around and moves away from the symmetry axis, but that re­orientation of adjacent N-terminal domains relative to each other also occurs (Supplementary Movie S1). The shift of C^α^ atoms in the RyR2^1–606^ structures docked into the closed and open RyR1 cryo-EM maps was on average 6–7 Å for the tested atoms of the exposed surface, but was only 4 Å for the atoms of the interfaces between domains (Supplementary Table S2). Docking of single domains into the maps of closed and open RyR1 suggests that the distance between individually docked A, B and C domains within a single monomer is longer, and the distance between individually docked domains of neighbouring monomers is shorter, than in 4jkq. Rearrangement of individual domains relative to each other upon channel gating may occur as well (Supplementary Movie S1).

**Figure 9:**
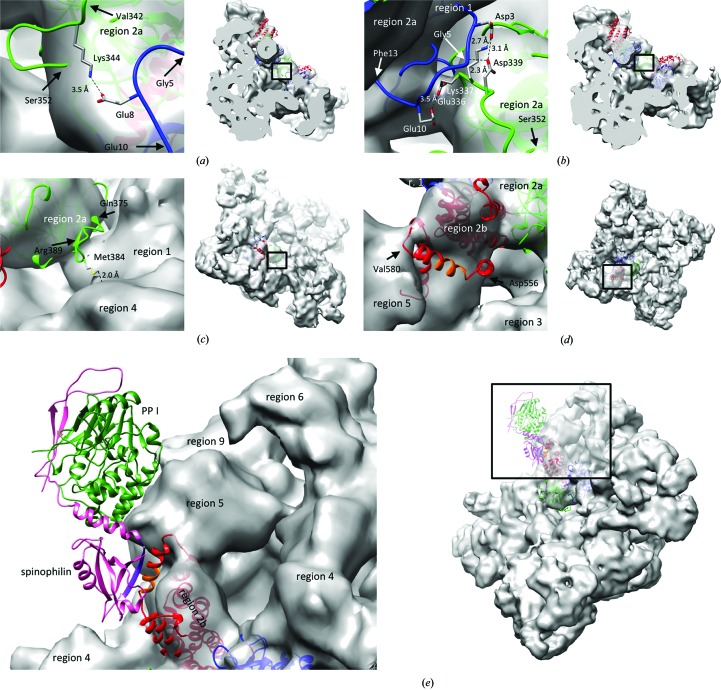
The hRyR2^1–606^ homology model docked into a cryo-EM map of the closed RyR1 (EMD 1606). (*a*, *b*) Side view of residues 5–10 (blue) and 342–352 (green) of a single monomer (*a*) and residues 1–12 (blue) and 336–342 (green) of two neighbouring monomers (*b*) pointing into the channel pore; (*c*) oblique view of the flexible loop (amino acids 377–387, green); (*d*) top view of the C-terminus (amino acids 545–606, red) containing the LIZ motif (amino acids 555–586) with the amino acids (L_565_EASSGIL) that interact with spinophilin shown in orange. (*e*) Oblique view of the above LIZ motif with attached complex of spinophilin (PDB entry 3egg chain *C*, pink; L_492_ELFPVEL, purple) with PP1 (PDB entry 3egg chain *A*, green). Domains A and C of hRyR2^1–606^ are shown in blue and red, respectively. RyR1 cryo-EM map regions are numbered according to Serysheva *et al.* (2008 ▶). In (*a*)–(*e*) the structural detail is shown in the left panel; a view of the whole RyR2 from the same angle is shown in the right panel. The rectangle corresponds to the area shown in the left panel.

Docking the homology model into the cryo-EM maps (Fig. 9 ▶) enabled examination of the missing residues relative to the neighbouring RyR domains. With the exception of residues 75–109, which formed the two previously predicted α-helices (Bauerová-Hlinková *et al.*, 2011 ▶), the residues absent in the A and B domains of 4jkq (amino acids 1–9 and 379–385) adopted a random-coil conformation in the model. The C-terminal part, encompassing amino acids 545–606, continued the armadillo motif of the C domain with three further α-helices. The N-terminal amino acids (1–9) pointed from the central rim into the channel pore, similar to amino acids 337–347, residues that were unresolved in previously published structures but present in the 4jkq structure. Residues 337–347 interact with residues 1–10 of the same monomer (Lys344 NZ–Glu8 OE2; Fig. 9 ▶ *a*) and of the neighbouring monomer (Lys337 NZ–Asp3 OD1, Asp339 OD1–Asp3 OD1, Asp339 OD1–Gly5 N, Glu336 OE2–Glu10 OE2; Fig. 9 ▶ *b*). The position of some residues suggested their interaction with other parts of the RyR molecules, since they pointed to or were contained in regions (numbered according to Serysheva *et al.*, 2008 ▶) of the cryo-EM map outside the central rim: the loop formed by residues 377–387 (Fig. 9 ▶ *c*) pointed from region 2a of the cryo-EM map into region 4, with a 2.0 Å distance between the map surface and CE of Met384. The C-terminal part of the model (amino acids 545–606), containing the leucine–isoleucine zipper (LIZ) motif (amino acids 555–586), was located on the surface of the cryo-EM map at the interfaces between regions 2b and 3 (Fig. 9 ▶ *d*) and between regions 2b and 5 (Fig. 9 ▶ *e*). Significantly, when the structure of the PP1–spinophilin complex (PDB entry 3egg; Ragusa *et al.*, 2011 ▶) was manually positioned to enable the previously observed interaction of the spinophilin (L_492_ELFPVEL) and RyR2 (L_565_EASSGIL) sequences (Marx *et al.*, 2001 ▶), the PP1–spinophilin complex closely followed the surface contour of regions 5 and 9 of the cryo-EM map (Fig. 9 ▶ *e* and Supplementary Movie S2). Spinophilin binds to hRyR2 predominantly by hydrophobic interactions, mainly between Leu565 and Leu572 of hRyR2 and Phe495 and Val497 of spinophilin.

### 3.8. Open *versus* closed structures   

The previously published structure of the N-terminal region of wild-type mRyR2 differs from that of the mRyR2 R420Q mutant in the relative position of individual domains (Kimlicka, Tung *et al.*, 2013  ▶). The different orientation of these domains was interpreted as a weakening of the interaction between domains A and C and a strengthening of the interactions between domains B and C in RyR mutants (Kimlicka, Lau *et al.*, 2013 ▶), by analogy to IP3Rs, in which the suppressor domain A decreases the ligand-binding affinity of the IP3Rs (Lin *et al.*, 2011 ▶; Seo *et al.*, 2012 ▶). To understand the possible effect of domain reorientation on RyR channel gating, it is important to distinguish precisely between the ‘open’ and ‘closed’ conformations of the RyR domains, which were presupposed to be analogous (Kimlicka, Lau *et al.*, 2013 ▶) to the ligand-bound and ligand-free conformations of the IP3 receptor domains, respectively (Seo *et al.*, 2012 ▶).

In the IP3R1 receptor, binding of InsP3 induces a change in the angles and distances of individual domains. To gauge the conformational changes upon RyR gating, we analysed the relative positions of the A, B and C domains of the known RyR and IP3R structures (Table 5 ▶). Parameters defining these positions for both IP3Rs and RyR2s, which were represented as inertia ellipsoids, are visualized in Supplementary Fig. S4. The ‘closed’ and ‘open’ RyR structures were approximated by docking individual hRyR2^1–606^ domains into the cryo-EM maps of RyR1 in the closed (EMD 1606) and open (EMD 1607) states, respectively (Samsó *et al.*, 2009 ▶). Differences between the ‘open’ and ‘closed’ conformations common to IP3R and RyR structures were observed only in the angle α, which was significantly larger in the closed than in the open conformation of both channels (Table 5 ▶). Significant differences between RyR structures could be observed in the angles of the minor axes of domains A and B (∠*a*_3_ *b*_3_), in the distances of domains A and B and of domains A and C, and also in the angle β. In all of the above measurements, none of the experimentally determined structures were significantly different from the ‘closed’ RyR structure but all were different from the ‘open’ RyR structure (Table 5 ▶).

The difference in the A-domain position relative to the C domain between the 2xoa and the ‘open’ structure on the one hand and the remaining RyR structures (4jkq, 4l4h and 4l4i; ‘closed’ structure) on the other results from a variance in the angle between the major axes of domains A and C (Table 5 ▶). This suggests that the 2xoa RyR1 structure might be partially open while all the RyR2 structures are in the closed state. The 4l4i and 4jkq structures differed from other experimentally determined structures (2xoa and 4l4h) by a small but significant variation in the angle (2°) between the minor axes of domains A and B. These values, however, were not significantly different from that of the ‘closed’ RyR structure, but they were significantly larger than the respective value for the ‘open’ RyR structure. These data suggest that all three structures of the RyR2 N-terminal region represent the closed state of the receptor.

## 4. Discussion   

In this study, we present the X-ray structure of the N-terminal part (amino acids 1–606) of human RyR2 at 2.38 Å resolution and its low-resolution structure in solution. We have also performed CD analysis of the RyR2 N-terminal region with the individual mutations P164S, R176Q, I419F and R420W that are responsible for inherited cardiac arrhythmias (http://www.fsm.it/cardmoc/) and of the I419F/R420W double mutant. For the I419F, R420W and I419F/R420W mutants, thermal stability curves were also measured. The hRyR2 N-terminal structure was docked into the cryo-EM map of the open and closed conformation of the native skeletal RyR1 isoform.

The recombinant hRyR2^1–606^ fragment was purified and crystallized at physiological pH and salt concentration (see §[Sec sec2]2). Importantly, the high chloride concentration previously reported to be crucial for solubility and sample homogeneity of mRyR2 (Kimlicka, Lau *et al.*, 2013 ▶) was not necessary for hRyR2^1–606^. The hRyR2^1–606^ X-ray structure provides new details that were not observed in the previously solved RyR N-terminal structures. The structure is in agreement with the results obtained by limited proteolysis of hRyR2^1–606^ fragments (Bauerová-Hlinková *et al.*, 2010 ▶), since the positions of loops encompassing Thr259 and Met348 are solvent-accessible.

The structure of individual domains in the RyR1 and RyR2 isoforms (PDB entries 2xoa, 4jkq and 4l4h) is almost identical but their relative positions showed substantial shifts as revealed by superposition of the C^α^ atoms of individual domains (Table 2 ▶). The direction of the shifts differed from those found in the structure of the IP3R N-terminal region upon InsP3 binding, as well as from those induced by docking into cryo-EM maps of open and closed RyRs (Table 5 ▶). Therefore, the observed main chain shifts most probably do not reflect N-terminal domain movements occurring during the process of channel gating. We attribute these shifts predominantly to differences in purification and crystallization conditions; both mRyR2^1–547^ (Kimlicka, Tung *et al.*, 2013 ▶) and oRyR1^1–559^ (Tung *et al.*, 2010 ▶) structures were obtained at a higher salt concentration (250 m*M* KCl and 1.2–1.5 *M* ammonium sulfate, respectively), which could produce these differences.

The mRyR2^1–547^ and hRyR2^1–606^ structures adopt a disparate arrangement of residues surrounding the previously proposed chloride-binding site (Kimlicka, Tung *et al.*, 2013 ▶). In hRyR2^1–606^ this site is comprised predominantly of Arg417, Arg276 and Tyr125 side chains that form a net of interdomain interactions that do not match those described for mRyR2^1–547^, but resemble those observed in oRyR1 (Tung *et al.*, 2010 ▶). Despite the presence of NaCl during purification and crystallization of hRyR2^1–606^, no chloride was observed in the vicinity of the aforementioned residues in hRyR2^1–606^, suggesting that the hRyR2 N-terminus has a rather low propensity to form the chloride-binding site in contrast to that observed for mRyR2^1–547^ (Kimlicka, Tung *et al.*, 2013 ▶). The presence or absence of the chloride ion in the experimental solutions did not affect the thermal stability of hRyR2^1–606^ as shown by thermal shift assay experiments (Borko *et al.*, 2013 ▶). Notably, the cytosolic chloride concentration of cardiac myocytes is low (∼23 m*M*; Akar *et al.*, 2003 ▶) and chloride-binding sites tend to have affinities in the millimolar range (for example a *K*_d_ of ∼0.6 m*M* in a neurotransmitter transporter homologue was observed; Tavoulari *et al.*, 2011 ▶). These data, together with our present observations indicating no evidence of an anion-binding site in the hRyR2 N-terminus, collectively suggest that the physiological importance and specificity of the proposed chloride-binding site in RyR2 (Kimlicka, Tung *et al.*, 2013 ▶) remains to be demonstrated.

The SAXS analysis in solution confirmed the overall shape of the molecule and provided useful information on the missing C-terminal ‘tail’ of the hRyR2^1–606^ structure (amino acids 545–606). Secondary-structure analysis of hRyR2^1–606^ and CD spectroscopy of hRyR2 N-terminal fragments of various lengths (Bauerová-Hlinková *et al.*, 2010 ▶, 2011 ▶), as well as the CD spectra presented here strongly suggest that the C-terminal tail is composed of α-helices that, however, were not folded into a globular shape. The last resolved residue of the crystal structure (Asn544) correlates with the last resolved residue of oRyR1^1–559^ (Gly532; PDB entry 2xoa, a difference of one amino acid), in which 24 C-terminal residues have similarly not been resolved. One possibility is that the tail may be cleaved by a protease, since there are a few protease cutting sites around residue 545 (*PeptideCutter*; Wilkins *et al.*, 1999 ▶). However, taking into account the stability of hRyR2^1–606^ in solution (Borko *et al.*, 2013 ▶), the proposal that the C-terminal part is removed specifically after protease cleavage appears implausible.

Alternatively, the unresolved C-terminal residues of hRyR2^1–606^ (545–606) may be present in the crystal but are not seen owing to inherent flexibility. This hypothesis is based on two observations: firstly, the shape of the SAXS envelope suggests that these residues form a tail in solution and, secondly, a cavity is present at the C-termini of the four symmetry-related neighbouring molecules in the crystal. The cavity has a cross-section of approximately 53 × 48 Å (Supplementary Fig. S5) that is large enough to accommodate the missing C-terminal tails, suggesting that the hRyR2^1–606^ molecule in the crystal is complete. Apparently the flexible tails form no additional contacts with either the globular part of the hRyR2^1–606^ structure or with themselves, resulting in a low and non-interpretable electron density in the cavity. A similar cavity can be observed also in the 2xoa structure.

Disulfide-bond formation has recently been shown to play an important role in homotetramer formation by the RyR2 N-terminal region (Zissimopoulos *et al.*, 2013 ▶), a property that is conserved in the IP3Rs (Zissimopoulos *et al.*, 2014 ▶). Of the ten cysteines in the structure of hRyR2^1–606^, only the SH groups of Cys47 and Cys132 are solvent-accessible. The homology model of hRyR2^1–606^ predicts a surface location for SH groups of two further cysteines: Cys548 and Cys577. Docking of the hRyR2 structure into the cryo-EM density map of RyR1 reveals that none of these cysteines are located at the intermolecular interfaces of the hRyR2^1–606^ homotetramer. Based on the hRyR2^1–606^ structure, we suggest that owing to their relative proximity, S—S bond formation could potentially occur only between Cys36 and Cys65 or between Cys131 and Cys158, since involvement of the remaining cysteines appears sterically unfeasible. Considering that these putative disulfide bonds are present within each subunit (*i.e.* intramolecular) and that such disulfides were empirically observed to stabilize the association between adjacent sub­units in the expressed N-terminal tetramer (Zissimopoulos *et al.*, 2013 ▶), this suggests that they may exert their effect on RyR2 oligomerization *via* an allosteric effect. Such an allosterically acting redox sensor of ryanodine receptor gating has been proposed previously (Pessah & Feng, 2000 ▶). This possibility could be tested by examining the effect of the above-mentioned cysteine mutants on the stability of RyR2 tetramerization.

Knowledge of the hRyR2^1–606^ structure gives us an opportunity to better understand structure–function relationships of several arrhythmogenic disease-associated mutations, including P164S, R176Q, I419F, R420W and the double mutant I419F/R420W (Supplementary Table S1). In the hRyR2^1–606^ structure, Pro164 has a standard conformation at the end of a β-strand. It is followed by a loop, and since its side chain is similar in size to that of serine, the P164S mutation should not cause any steric clashes in this region. Arg176 is located in a loop; its main-chain O atom makes several hydrogen bonds to main-chain N atoms of neighbouring residues and its guanidine group points into solution, forming a stacking interaction with that of Arg169. Its replacement with glutamine in the R169Q mutant diminishes the size of the side chain, reduces the positive charge and removes the stacking interaction. In contrast, Arg176 is located on the surface of hRyR2^1–606^; its mutation should not cause structural changes. The effect of the above mutations will be better understood only in the context of the interfacing residues of the whole RyR molecule, which are currently not known.

Ile419 is located in the central α-helix. It is buried and forms contacts with Thr415, Ala416, Leu488, Glu492, Phe489 and Met494 (Table 4 ▶). Replacement of Ile with the bulkier Phe residue results in profound steric clashes with neighbouring residues in all rotamers, and this is consistent with the decreased thermal stability of the mutated hRyR2 fragment and therefore may be the cause of the severe cardiac dysfunction in the I419F mutant.

Secondary-structure prediction indicates that the central helix may undergo a structural change upon the R420W mutation. The decrease in α-helicity observed in CD spectra for the R420W mutant supports this prediction. However, the side chain of Arg420 is partially solvent-accessible; it is therefore likely that the tryptophan side chain in the R420W mutant adopts a conformation pointing to the solvent, thus avoiding significant structural changes (Fig. 6 ▶ *c*). This is supported by the unchanged thermal stability of R420W with respect to that of the wild type (Fig. 7 ▶ *a*) and the R420Q mutant (Kimlicka, Tung *et al.*, 2013 ▶). Arg420 is not conserved between the RyR isoforms, but it is well conserved among RyR2s from different organisms. The existence of two different (W/Q) mutations of Arg420 also indicates its potentially important role.

We have shown by docking of the hRyR2^1–606^ model into a cryo-EM map of RyR1 (Samsó *et al.*, 2009 ▶) that the LIZ motif involved in binding of spinophilin–PP1 (Marx *et al.*, 2001 ▶) is most likely to be exposed on the surface of the valley formed between RyR regions 2b and 5 (Serysheva *et al.*, 2008 ▶), and that spinophilin and PP1 (PDB entry 3egg; Ragusa *et al.*, 2011 ▶) adopt a specific conformation in which the PP1 surface topology accurately complements that of the RyR surface. Elucidating the structure of the RyR2–PP1 complex would be important to help unravel the molecular mechanism of RyR2 dephosphorylation. Understanding the structure and function of this fundamental regulatory process in muscle excitation–contraction coupling may be vital in delivering therapeutic strategies for the prevention of hRyR2 phosphorylation-related myocardial pathologies, such as mutation-induced and heart failure-induced tachyarrhythmias and sudden cardiac death.

## 5. Accession number   

Coordinates and structure factors for the hRyR2^1–606^ structure reported in this paper have been deposited in the RCSB Protein Data Bank with accession code 4jkq.

## 6. Related literature   

The following references are cited in the Supporting Information for this article: Kawamura *et al.* (2013 ▶), Krissinel & Henrick (2007 ▶), Meli *et al.* (2011 ▶), Pace *et al.* (1995 ▶), Stewart *et al.* (2003 ▶) and Tester *et al.* (2012 ▶).

## Supplementary Material

PDB reference: N-terminal region of the human ryanodine receptor 2, 4jkq

Supporting Information. DOI: 10.1107/S1399004714020343/lv5073sup1.pdf

Supplementary Movie S1.. DOI: 10.1107/S1399004714020343/lv5073sup2.mp4

Supplementary Movie S2.. DOI: 10.1107/S1399004714020343/lv5073sup3.mp4

## Figures and Tables

**Table 1:** Structure solution and refinement Values in parentheses are for the outer shell.

Resolution range† ()	44.772.39 (2.4492.39)
Completeness† (%)	99.4
Cutoff	2.0
No. of reflections, working set	27960 (1598)
No. of reflections, test set	1444 (74)
Final *R*_cryst_	0.224 (0.336)
Final *R*_free_	0.261 (0.334)
Coordinate ESU based on *R*/*R*_free_ ()	0.316/0.240
Cruickshank DPI	0.316
No. of non-H atoms	
Protein	3934
Ligand	0
Water	79
Total	4013
R.m.s. deviations ()	
Bonds ()	0.008 (0.019)
Angles ()	1.185 (1.946)
Chiral centres (^3^)	0.082 (0.200)
Planar groups ()	0.004 (0.020)
Average *B* factors (^2^)	
Protein	37.7
Main chain	36.9
Side chain	38.6
Water	47.2
Wilson plot *B* factor (^2^)	50.4
Ramachandran plot	
Most favoured (%)	93.1
Additionally allowed (%)	6.1
Outliers (%)	0.8

† From Borko *et al.* (2013 ▶).

**Table 2:** Superposition of hRyR2^1606^ (PDB entry 4jkq) with mRyR2^1547^ (PDB entry 4l4h) and oRyR1^1559^ (PDB entry 2xoa) based on C atoms present in all three structures Column headings indicate parts of the sequences (whole chains, AA, BB or CC) used for superposition, for which only C atoms 2 apart were used; row headings indicate parts of the sequences (whole chains, A/A, B/B/ or C/C), for which an r.m.s.d. was calculated. R.m.s.d. and maximal deviations were calculated from all C atoms of equivalent residues present in all three structures. Assignment of domains (hRyR2 numbering): A, 10223; B, 224408; C, 409544.

	Superposition of 4jkq and 4l4h				Superposition of 4jkq and 2xoa			
		Domains				Domains		
	Whole chains	AA	BB	CC	Whole chains	AA	BB	CC
R.m.s.d. ()								
Whole chains	**2.1**	3.5	2.7	2.9	**2.2**	2.6	2.6	2.5
A/A	1.7	**0.8**	2.9	3.5	1.3	**1.0**	2.6	2.6
B/B	3.0	4.5	**2.9**	3.1	3.2	3.7	**3.1**	3.1
C/C	1.1	4.4	2.0	**0.4**	1.1	2.6	1.6	**0.8**
Rotation angle between superposed structures ()	0.7	6. 7	3.7	4.6	0.6	4.1	3.3	2.5
Maximum deviation ()	24.0	3.8	23.7	2.5	24.5	4.9	24.4	8.4
Most deviating residue (hRyR2 numbering)	Gly348	Asn198	Gly348	Glu473	Gly348	Ala223	Gly348	Ala440
No. of superposed atoms	387	157	150	112	403	158	144	107

**Table 3:** Interfaces between RyR N-terminal domains A, B and C

	4jkq			4l4h			2xoa		
Interface	A/B	A/C	B/C	A/B	A/C	B/C	A/B	A/C	B/C
Interface area† ‡ (^2^)	812	475	561	761	323	576	758	429	557
No. of residues at the interface	23/24	14/14	18/16	25/24	10/14	14/18	24/21	14/14	17/15
Surface complementarity	0.69	0.65	0.57	0.58	0.67	0.48	0.58	0.75	0.63
Hydrogen bonds‡	5	7 (4)	6 (5)	8	8 (5)	8 (6)	7	9	9
Salt bridges‡	3	7 (4)	3 (2)	1	1	3	1	3	3
*G* of interaction ‡ (kcalmol^1^)	5.2	0.2 (+1.4)	1.9 (1.6)	8.0	0.2	0.8	6.4	1.4	3.4

† The assignment of domains is as follows. oRyR1: A, 1205, B, 206395; C, 396532. hRyR2, mRyR2: A, 1223; B, 224408; C, 409544.

‡ Determined by *PISA* (http://www.ebi.ac.uk/msd-srv/prot_int/cgi-bin/piserver). Only hydrogen bonds of 3.5 were accepted. Values for interaction between the central helix and domains A and B are in parentheses.

**Table 4:** Charged, polar and hydrophobic contacts of the central helix residues Central helix residues (column I) forming charged, polar and hydrophobic contacts with residues from domains A, B and C (column II). Abbreviations: ARVD2, arrhythmogenic right ventricular dysplasia type 2; CPVT1, catecholaminergic polymorphic ventricular tachycardia type 1; E-IVF, idiopathic ventricular fibrillation induced by emotion or exercise; SCD, sudden cardiac death associated with drowning; SUO, syncope of unknown origin.

I	II	Distances ()	Mutation/disease†	Comment
Glu411	Arg485	3.5		PSA‡
Glu412	Arg235, Asn409	3.5		B/C interface
Arg414	Tyr125	3.5	R414C, L/SCD, CPVT1, E-IVF, SUO	A/C interface
Thr415	Tyr462, Glu410	3.5	T415R/CPVT1	Buried
Arg417	Asp61, Glu40, Arg420	3.5		A/C interface
Ile419	Phe489, Met494, Thr415, Ala416, Leu488, Glu492§	4.5	I419F/SCD, SUO	B/C interface
Arg420	Val300, Ala298, Arg417	3.5	R420Q, W/CPVT1, ARVD2	B/C interface
Ser421	Glu40	3.5		A/C interface
Phe426	Thr422, Val452, Leu456, Leu459, Leu497, Cys501, Leu553	4.5		buried
Arg428	Asp446	3.5		PSA
Phe429	Leu433, Val452, Leu447, Ile449, Cys501, Leu505	4.5		Buried
Leu433	Phe429, Phe415, Arg504§, Leu505	4.5	L433P/ARVD2	Buried
Asp434	Arg504, Lys438	3.5		PSA
Leu436	Ala442, Val445, Leu447, Val517, Ala518	4.5		PSA
Lys438	Asp434	3.5		PSA

† http://www.fsm.it/cardmoc (Bauce *et al.*, 2002 ▶; Choi *et al.*, 2004 ▶; Creighton *et al.*, 2006 ▶; Medeiros-Domingo *et al.*, 2009 ▶; Nishio *et al.*, 2006 ▶; Tester *et al.*, 2004 ▶, 2005 ▶; Tiso *et al.*, 2001 ▶).

‡ Partially solvent accessible.

§ Hydrophobic part of the side chain.

**Table 5:** Analysis of spatial relationships between domains of the N-terminal RyR and IP3R structures

	RyR						IP3R	
	2xoa	4jkq	4l4h	4l4i	Domains A, B and C docked into EMD 1606	Domains A, B and C docked into EMD 1607	InsP3, free (3uj4, 3t8s chain A )	InsP3, bound (3uj0, 3t8s chain B)
Angle of axes† ()								
*a*_1_ *b*_1_	46.7	49.1	56.3	56.5	51.8	51.6	76.3 0.8	82.2 1.6‡
*a*_1_ *c*_1_	41.9[Table-fn tfn10]	45.8	46.3	46.9	46.1	40.2[Table-fn tfn10]	59.7 1.1	54.2 3.1
*a*_2_ *b*_2_	92.1	90.6	88.0	85.8	89.9	91.0	42 1.73	51.1 0.46‡
*a*_3_ *b*_3_	87.1[Table-fn tfn11]	84.8	86.5[Table-fn tfn11]	85.0	85.0	80.9[Table-fn tfn12]	61.7 1.1	62.8 1.1
*b*_3_ *c*_3_	85.5[Table-fn tfn12]	94.8	94.0	93.4	95.2	95.2	73.0 2.7	83.6 1.2‡
Distance between centres of gravity ()								
AB	31.1	31.7	31.3	31.3	32.4	36.0[Table-fn tfn12]	35.2 0.1	35.0 0.1
AC	30.3	30.5	30.7	30.6	30.9	37.5[Table-fn tfn12]	28.8 0.3	29.1 0.02
BC	30.6	30.4	30.9	30.5	33.2	32.6	31.2 0.1	29.8 0.1‡
Angle[Table-fn tfn13] ()								
	59.9	58.4	59.7	58.9	63.3	52.7[Table-fn tfn12]	57.7 0.4	54.2 0.4‡
	58.8	58.9	59.3	59.3	56.1	66.0[Table-fn tfn12]	51.2 0.7	52.4 0.1
	61.3	62.7	61.0	61.7	60.6	61.3	71.1 0.4	73.3 0.4‡
State	Closed	Closed	Closed	Closed	Closed	Open	Closed	Open

† *a*, *b*, *c* are the axes of the ellipsoids approximating domains A, B and C, respectively. The numbers 1, 2 and 3 correspond to the major, medium and minor axes of the ellipsoids (see Supplementary Fig. S4 for an illustration).

‡ Different from the InsP_3_-free structures (*p* 0.05, *t*-test).

§ Different from 4jkq, 4l4h, 4l4i and ‘closed’ RyR2^1606^ (*p* 0.05, one-way ANOVA).

¶ Different from 4jkq and 4l4i (*p* 0.05, *t*-test).

†† Different from the remaining RyR structures (*p* 0.05, one-way ANOVA).

‡‡ , , are the angles BAC, ABC, and ACB of the triangle ABC formed by the centres of the ellipsoids approximating domains A, B and C, respectively.

## Footnotes

1 Supporting information has been deposited in the IUCr electronic archive (Reference: LV5073).
