# Supplementary material for: Structural insights into the human RyR2 N-terminal region involved in cardiac arrhythmias
Source: Acta Crystallogr D Biol Crystallogr. 2014 Oct 23;70(Pt 11):2897–912. doi: 10.1107/S1399004714020343 (PMC4220973; doi:10.1107/S1399004714020343)
Supplement: Supplementary file 1 [file d-70-02897-sup1.pdf]

Acta Crystallographica Section D

Volume 70 (2014)

Supporting information for article:

Structural insights into the human RyR2 N-terminal region involved in cardiac arrhythmias

Ľubomír Borko, Vladena Bauerová-Hlinková, Eva Hostinová, Juraj Gašperík, Konrad Beck, F. Anthony Lai, Alexandra Zahradníková and Jozef Ševčík

S1. Supplemental Methods

S1.1. Determination of the solution structure by SAXS

After preparative SEC (Superdex 200 prep. grade 16/60, GE Healthcare) the sample was concentrated to ≈2.5 $mg.ml^{-1}$ with Microsep Advance and Nanosep centrifugal devices (Pall Corporation) with 10 kDa cutoffs, reanalyzed by SEC (Superose 12 10/30, GE Healthcare) and tested by DLS after 12, 24, and 48 hours (Borko *et al.*, 2013). Five days after the last DLS measurement, the sample was analyzed by analytical SEC (Superose 12 10/30, GE Healthcare) to double-check its quality and stability. SEC data showed high homogeneity of the sample immediately before and 24 hours after DLS testing. DLS data showed adequate stability of the sample over at least four days (Borko *et al.*, 2013). The sample was concentrated to ≈16 $mg.ml^{-1}$ and diluted at ratios of 1:1 to 1:5. The concentration of each diluted sample was measured by a Nanodrop device (Thermo Scientific) using $A_{0.1\%,280nm,\ 1cm}$ = 1.1. No radiation damage was detected. Beam line characteristics for SAXS measurements are shown in Table S3. SAXS data were measured at four different concentrations (Table S4). The scattering curve of the particle was obtained by averaging eight measurements and subtracting average buffer scattering from sample scattering. There was a dependence of R_g on sample concentration (Table S4) indicating inter-particle interactions, and therefore R_g values were extrapolated to infinite dilution.

The difference between R_g values calculated from Guinier approximation and from the P(r) function might indicate a slight heterogeneity of the sample, which correlated with the 31.2% polydispersity of the monomeric peak (Borko *et al.*, 2013). Adequate protein folding was indicated by the Kratky plot. The Porod volume of the particle was ≈152 000 $Å^3$, which gave a molecular weight of ≈76 kDa. The difference of 8 kDa from the expected $hRyR2^{1-606}$ MW of 67.8 kDa likely indicates shape heterogeneity caused by flexible regions of the protein. The position of the maximum of the pair distribution function at a short distance and the extended tail of the function indicate that some segments of the structure might be extended.

The solution structure of $hRyR2^{1-606}$ was determined by *ab initio* modeling with *GASBOR* using simulated annealing. All created models had a similar shape. The model with χ^2 and NSD values of 0.95 and 1.538, respectively, was chosen for further analysis.

S1.1. Determination of relative domain orientation

Previous measurements of relative domain orientation in the IP3R1 were performed relative to the long α-helix in the suppressor domain (equivalent to domain A of the RyR), which is not present in RyR1 and not resolved in RyR2. To obtain an unequivocal representation of the domain positions, individual domains were represented by the CA-atoms of residues present in all published structures and in 4JKQ, and their position and shape were characterized by their inertia ellipsoids in *CHIMERA*. (Figure S4). To determine, which of the characteristics of inertia ellipsoids were significantly changed

in IP3R1 upon binding of inositol trisphosphate, a similar calculation was performed for the equivalent domains of all chains of the known IP3R1 N-terminal structures (3T8S, 4UJ0, 4UJ4; three Ins_3P-bound and three Ins_3P-free structures). It was assumed that the ligand-free structure represents the closed channels and the ligand-bound structure represents the open channel. To estimate the changes occurring in RyR channels, the A, B, C domains of 4JKQ independently docked into cryoEM maps of the RyR1 in the closed and open state (Samso *et al.*, 2009) were analyzed in the same manner.

S1.2. Docking the crystal structure and the model into cryo-electron microscopy maps.

The crystal structure as well as the model could be docked into electron density maps of the RyR1 channel (EMD 1275, 1606, 1607, 1274, 5014). The *ADP_EM* and *SITUS* software packages provided essentially identical positions, similar to those previously reported for the 2XOA structure of $oRyR1^{1\text{-}559}$ (Tung *et al.*, 2010). Docking contrasts were not significantly different between *ADP_EM* and *SITUS*. Since the RMSD between the symmetrical copies of the first four best fits was much smaller in *SITUS* (0.063 ± 0.020 and 0.069 ± 0.021 Å for crystal structure and model, respectively) than in *ADP_EM* (4.11 ± 0.25 and 3.43 ± 0.28 Å for crystal structure and model, respectively), *SITUS* results were analyzed further. The cross-correlation coefficients of the second-best symmetrically independent fit were 0.52 ± 0.04 of the best fits for both, crystal structure and model, indicating a sufficiently high docking contrast. For further analysis we selected the maps of the RyR1 channel in the open and closed conformation, prepared in parallel under comparable conditions (Samso *et al.*, 2009). The RMSD between $hRyR^{1\text{-}606}$ docked into the map of the closed and the open RyR1 were 4.92 and 4.72 Å for crystal structure and model, respectively. When individual A, B, C domains of $hRyR2^{1\text{-}606}$ were independently docked into the cryoEM maps, their optimal position was very close to, but not identical with the positions of the corresponding parts of the whole crystal structure (RMSD of 1.65 and 1.39 Å in the closed and open state, respectively).

S1.3. CD spectroscopy

Solution secondary structure and thermal stability of $hRyR2^{1\text{-}606}$ wild type and mutant proteins were assessed by far-UV CD spectroscopy using 1-nm bandwidth, 0.2-nm steps, 3 to 5 s per point accumulation time. Prior to measurements, samples were centrifuged (1h, 14 000×g, 4°C). The buffer was the same as used for crystallization of the wild type protein except that NaCl was replaced by NaF for increased transparency. Spectra were recorded from 260 to ≤ 190nm at 4°C, and from 20°C on in 5°C intervals up to precipitation indicated by a steep rise in the dynode voltage. Protein concentration was determined from the absorbance at 280 nm assuming extinction coefficients as calculated from the amino acid composition (Pace *et al.*, 1995).

S1.4. Information about macromolecular production, crystallization and data collection and processing.

Production, crystallization and data collection have been described previously (Borko *et al.*, 2013). Relevant details are summarized in Table S5, S6 and S7.

S2. Multimedia Files

Supplementary Movie S1 Docking of the 4JKQ structure and its domains A, B, C into RyR cryo-EM maps. Top view (from the cytoplasm into SR lumen) and side view of pseudo-atomic models of the 4JKQ structure of hRyR2$^{1-606}$ (domains A, B, C shown in blue, green and red, respectively) docked into the cryo-EM maps of the ryanodine receptor in the closed (EMD 1606) and open state (EMD 1607). Top view of the 4JKQ structure and its domains A, B, C docked into the cryo-EM maps EMD 1606 and EMD 1607. Movement of the A, B, C domains of the 4JKQ structure upon channel opening. Domains A, B, C are shown in blue, green and red, respectively

Supplementary Movie S2 Docking of spinophilin and protein phosphatase I on the LIZ of hRyR2$^{1-606}$. Domains A, B, C of hRyR2$^{1-606}$ are shown in blue, green and red, respectively. Spinophilin (PDB 3EGH chain C) and phosphoprotein phosphatase I (PDB 3EGH chain A) are shown in beige and light green, respectively. The motifs LELFPVEL in sphinophilin and LEASSGIL in RyR2 (see main text) are shown in pink and purple, respectively.

Supplementary References

Borko, L., Kostan, J., Pevala, V., Gasperik, J., Hostinova, E., Urbanikova, L., Zahradnikova, A., Carugo, K. D., Hlinkova, V. B. & Sevcik, J. (2013). *Protein Pept Lett.* **20**, 1211-1216.

Emsley, P. & Cowtan, K. (2004). *Acta Cryst D* **60**, 2126-2132.

Kawamura, M., Ohno, S., Naiki, N., Nagaoka, I., Dochi, K., Wang, Q., Hasegawa, K., Kimura, H., Miyamoto, A., Mizusawa, Y., Itoh, H., Makiyama, T., Sumitomo, N., Ushinohama, H., Oyama, K., Murakoshi, N., Aonuma, K., Horigome, H., Honda, T., Yoshinaga, M., Ito, M. & Horie, M. (2013). *Circ J* **77**, 1705-1713.

Krissinel, E. & Henrick, K. (2007). *J Mol Biol* **372**, 774-797.

Medeiros-Domingo, A., Bhuiyan, Z. A., Tester, D. J., Hofman, N., Bikker, H., van Tintelen, J. P., Mannens, M. M., Wilde, A. A. & Ackerman, M. J. (2009). *J Am Coll Cardiol* **54**, 2065-2074.

Meli, A. C., Refaat, M. M., Dura, M., Reiken, S., Wronska, A., Wojciak, J., Carroll, J., Scheinman, M. M. & Marks, A. R. (2011). *Circ Res* **109**, 281-290.

Pace, C. N., Vajdos, F., Fee, L., Grimsley, G. & Gray, T. (1995). *Protein Sci* **4**, 2411-2423.

Samso, M., Feng, W., Pessah, I. N. & Allen, P. D. (2009). *PLoS Biol* **7**, e85.

Stewart, R., Zissimopoulos, S., Lai, F.A. (2003). *Biochem J* **376**, 795-799.

Tester, D. J., Medeiros-Domingo, A., Will, M. L., Haglund, C. M. & Ackerman, M. J. (2012). *Mayo Clin Proc* **87**, 524-539.

Tung, C. C., Lobo, P. A., Kimlicka, L. & Van Petegem, F. (2010). *Nature* **468**, 585-588.

Table S1 Selected mutations of the hRyR2$^{1\text{-}606}$ associated with cardiac diseases.

Mutation/Disease*	Position**	Effect#	Interactions of the side-chain in wt molecule
L62F/CPVT	A/B interface<br>loop	2	T52,S285,V282,C36,P60
P164S/CPVT, SCD	A (partially solvent accessible)<br>β-strand	1, 4	Y115,R169
R169Q/CPVT	A (partially solvent accessible)<br>loop	2, 3	D179,R176,Y115
V186M/CPVT	A (partially solvent accessible)<br>loop	2	W159,T161,A118,E156
I217V/SUO	A/B interface<br>β-strand	2	V21,V282,E19,S285
E243K/CPVT	B (partially solvent accessible)<br>β-strand	2, 3	R389,H238,M241
F329L/CPVT	B (buried)<br>β-strand	2	L306,I363,L314,M393,H365
R332W/CPVT	B (buried)<br>β-strand	2,3,4	E345, S334,W371,T330,L338,N364
D400H/SCD	A/B interface<br>loop	2, 3	Y374,M241
R414C/CPVT	A/C interface<br>α-helix	2, 3, 4	Y125,Y462
R414L/SCD SUO	A/C interface<br>α-helix	2, 3, 4	Y125,Y462
T415R/CPVT	C (buried)<br>α-helix	2, 3	Y462,E411,F489,L488,I419,R485,E410
I419F/SCD SUO	B/C interface<br>α-helix	2	F489,V423,A271,A416,L488,E492, M494,T415
R420W/ARVD2	B/C interface<br>α-helix	2, 3, 4	V300,R417,R298,F424,G303
R420Q/CPVT	B/C interface<br>α-helix	2, 3	V300,R417,R298,F424,G303
L433P/ARVD2	C (buried)<br>α-helix	2	F429,F514,R504,L505,L447

* http://www.fsm.it/cardmoc, (Medeiros-Domingo *et al.*, 2009, Tester *et al.*, 2012, Kawamura *et al.*, 2013, Meli *et al.*, 2011)

** Analysis was performed by PISA server (Krissinel & Henrick, 2007); elements of the secondary structure were assigned according to *COOT* (Emsley & Cowtan, 2004)

Possible effects of mutations are based on change of conformation of: main chain (1), side chain size (2), charge (3), polarity (4).

Polar and charged interactions were considered up to 3.5 Å, hydrophobic contacts up to 4.5 Å.

Abbreviations: ARVD2 – arrhythmogenic right ventricular dysplasia type 2; CPVT1 – catecholaminergic polymorphic ventricular tachycardia type 1; SCD – sudden cardiac death associated with drowning; SUO – syncope of unknown origin

Table S2 Difference between positions of and distances between CA atoms of $hRyR2^{1-606}$ models docked into the closed (EMD 1606) and open conformation (EMD 1607) of the RyR

	Parameter	Difference between structures docked into EMD 1606 and 1607		No. of tested CA atoms/atom pairs
		4JKQ structure	Domains A, B, C docked separately	
Distances between pairs of adjacent CA atoms in neighboring monomers (Å)	Average<br>Maximum increase<br>Maximum decrease	2.8 ± 0.5<br>8.2<br>-1.3	3.1 ± 0.6<br>7.8<br>-2.7	23
Positions of CA atoms of surface exposed residues (Å)	Average<br>Maximum	6.2 ± 0.4<br>8.4	6.7 ± 0.5<br>9.6	21
Positions of CA atoms of the ABC interface (Å)	Average<br>Maximum	4.4 ± 0.1<br>6.6	4.3 ± 0.2<br>7.4	56
Distances between pairs of adjacent CA atoms from domains A/B (Å)	Average<br>Maximum increase<br>Maximum decrease	N/A	0.2 ± 0.2<br>1.7<br>-1.2	18
Distances between adjacent CA atoms from domains A/C (Å)	Average<br>Maximum increase<br>Maximum decrease	N/A	0.14 ± 0.06<br>0.6<br>-0.4	18
Distances between adjacent CA atoms from domains B/C (Å)	Average<br>Maximum increase<br>Maximum decrease	N/A	0.1 ± 0.4<br>1.2<br>-1.0	6

Distance difference is expressed as the distance between a pair of adjacent CA atoms from two domains in structures docked into EMD1607 minus the distance between the same pair of CA atoms in structures docked into EMD 1606.

Table S3 Beamline characteristics

Optics	
Source	Bending Magnet
Monochromator	Horizontal focusing triangular Si (111) asymmetric cut 7°
Mirror	Rhodium coated flat mirror on Zerodur substrate with gravimetrical bending
Beam size at the detector	2 × 0.6 mm^2
Wavelength	0.15 nm
Flux at sample	5 × 10^{11} ph/s/100mA
Sample to detector distances	Standard: 2.7 m
Resolution SAXS @ 2.7 m	From s = 0.06 nm^{-1} to 6 nm^{-1}
Exposition time	15 s
Sample stage	
Vacuum cell	30 µl volume, automated filling and rinsing; temperature: 10°C
Small automated sample changer	Sample volume 70-100 µl
Detector	
2D Photon counting Pilatus 1M-W pixel	0.172 mm pixel size, 67 × 420 mm^2 useful area, 3 ms

X-ray detector	frame rate

Table S4 Concentration dependence of SAXS measurements

c (mg/ml)	R_g (Å)*	R_g (Å)**	I(0)	No. of Guinier points	D_{max} (Å)	V (Å^3)
8.1	39.6 ± 1.5	42.5	67.23	43-89 (47)	138.9	169 290
6	38.9 ± 0.2	41.5	70.87	35-91 (57)	136.5	161 840
4.6	37.6 ± 0.3	40.1	65.49	43-95 (53)	131.7	154 550
3.9	37.1 ± 0.3	39.4	62.01	34-96 (63)	130.0	151 880
0***	36.9 ± 0.3	40.1	68.69	34-97 (64)	135.0	152 180

*Rg from the Guinier approximation, **Rg from the P(r) function, ***extrapolation to zero concentration

c – concentration, calculated from the absorbance at 280nm and A0.1%,280nm, 1cm = 1.1 mg/ml

Rg – radius of gyration

I(0) – scattering intensity at 0°

Guinier points – linearity region for Rg estimation, number of Guinier points in parenthesis.

Dmax – maximum dimension of the particle

V – volume of the particle

Table S5 Macromolecule production information

Source organism	Homo sapiens
DNA source	Plasmid BT4 (Stewart *et al.*, 2003)
Forward primer	5’CCCCATGGCCGATGGGGGCGAGGGCGAA
Reverse primer	5’CCGGATCCTTA*GTGATGATGATGATGATG*TCTTCCATGTTTGTCTAAAAGTGAGATAATAG
Cloning vector	pET28a
Expression vector	pET28a
Expression host	*Escherichia coli*
Complete amino acid sequence of the construct produced	MADGGEGEDEIQFLRTDDEVVLQCTATIHKEQQKLCLAAEGFGNRLCFLESTSNSKNVPPDLSIC<br>TFVLEQSLSVRALQEMLANTVEKSEGQVDVEKWKFMMKTAQGGGHRTLLYGHAILLRHSYSG<br>MYLCCLSTSRSSTDKLAFDVGLQEDTTGEACWWTIHPASKQRSEGEKVRVGDDLILVSVSSERY<br>LHLSYGNGSLHVDAAFQQTLWSVAPISSGSEAAQGYLIGGDVLRLLHGHMDECLTVPSGEHGEE<br>QRRTVHYEGGAVSVHARSLWRLETLRVAWSGSHIRWGQPFRLRHVTTGKYLSLMEDKNLLLM<br>DKEKADVKSTAFTFRSSKEKLDVGVRKEVDGMGTSEIKYGDSVCYIQHVDTGLWLTYQSVDVK<br>SVRMGSIQRKAIMHHEGHMDDGISLSRSQHEESRTARVIRSTVFLFNRFIRGLDALSKKAKASTV

	DLPIESVSLSLQDLIGYFHPPDEHLEHEDKQNRLRALKNRQNLFQEEGMINLVLECIDRLHVYSSA AHFADVAGREAGESWKSILNSLYELLAALIRGNRKNCAQFSGSLDWLISRLERLEASSGILEVLH CVLVESPEALNIIKEGHIKSIISLLDKHGR

NcoI and BamHI restriction sites are denoted by single and double underline, respectively; His6-tag is denoted by italics

Table S6 Crystallization

Method	Vapor diffusion, hanging drop
Plate type	24-well Linbro plate
Temperature (K)	295
Protein concentration	5 mg.ml $^{-1}$
Buffer composition of protein solution	20 mM Tris-HCl, pH 7.5, 150 mM NaCl, 10 % glycerol, 7 mM 2-mercaptoethanol, 1 mM CHAPS, 0.1 % betaine
Composition of reservoir solution	20 m*M* Tris-HCl, 150 m*M* NaCl, 10% glycerol, 7mM 2-mercaptoethanol, 1 m*M* chaps, 0.1% betaine, 100 m*M* HEPES, 200 m*M* ammonium formate, 21% PEG 3350, pH 8.0
Volume and ratio of drop	3µl; 2:1
Volume of reservoir	400 µl

Table S7 Data collection and processing

Values for the outer shell are given in parentheses.

Diffraction source	Bessy beamline 14.1
Wavelength (Å)	0.918
Temperature (K)	100
Detector	Marmosaic 225 mm CCD area detector
Crystal-detector distance (mm)	290.16
Rotation range per image (°)	0.5
Total rotation range (°)	180
Exposure time per image (s)	6
Space group	$P4_22_12$
a, *b*, *c* (Å)	75.45, 75.45, 248.84
α, β, γ (°)	90, 90, 90
Mosaicity (°)	1.14
Resolution range (Å)	124.42–2.39 (2.52–2.39)
Total No. of reflections	406071
No. of unique reflections	29469
Completeness (%)	100 (95)
Redundancy	13.8
< I/σ(I) >	12.2 (2.0)
$R_{r.i.m.}$†	0.166

† Estimated $R_{r.i.m.} = R_{merge}[N/(N-1)]^{1/2}$, where N = data multiplicity.

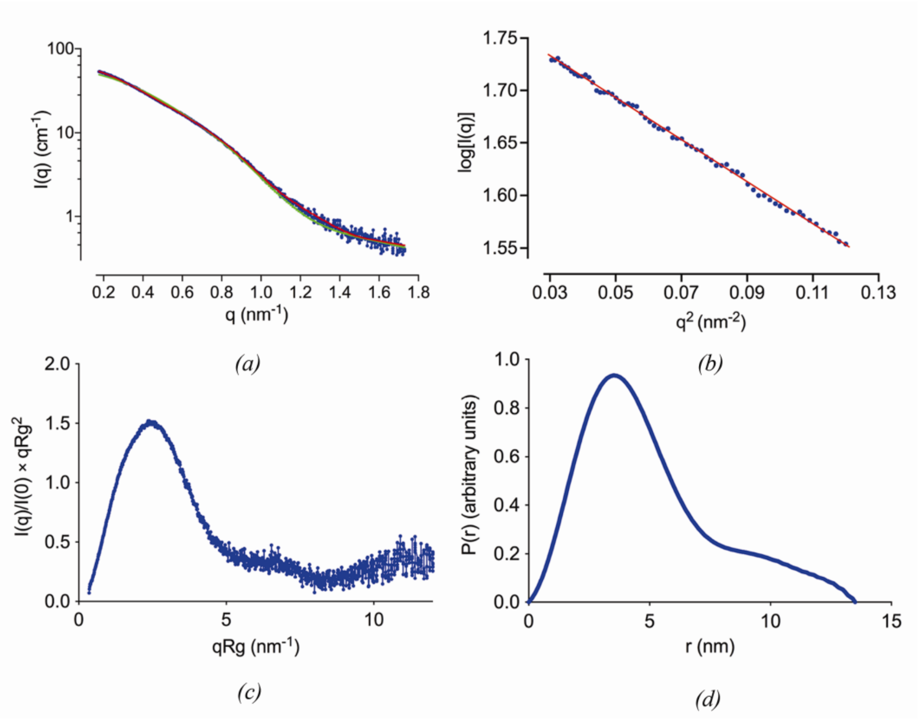

Figure S1 Representative plots produced during hRyR2$^{1\text{-}606}$ SAXS data processing. (a) Scattering plot in log Y scale. Blue – data points, red – GASBOR fit of the chosen model, green – CRYSOL fit of the I-TASSER model; (b) Guinier plot, red line represents guinier region, sRg limits: 0.628 – 1.288; (c) Normalized Kratky plot; (d) P(r) plot.

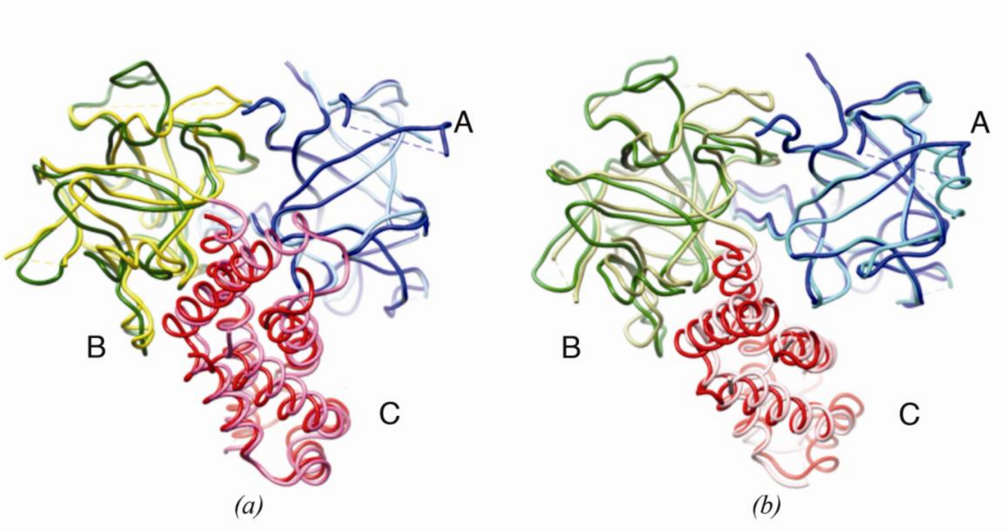

Figure S2 N-terminal structures of the RyR. Least-squares superposition of N-terminal structures (a) hRyR2$^{1\text{-}606}$ and mRyR2$^{1\text{-}547}$, (b) hRyR2$^{1\text{-}606}$ and oRyR1$^{1\text{-}559}$. Superposition was based on CA atoms of domain A (aa 1-223, hRyR2 numbering); only atoms with RMSD less than 2Å were included into calculation. Domain colors: hRyR2$^{1\text{-}606}$ A-blue, B-green, C-red; mRyR2$^{1\text{-}547}$ A-light blue, B-yellow, C-magenta, oRyR1$^{1\text{-}559}$ A-turquoise, B-khaki, C-light pink

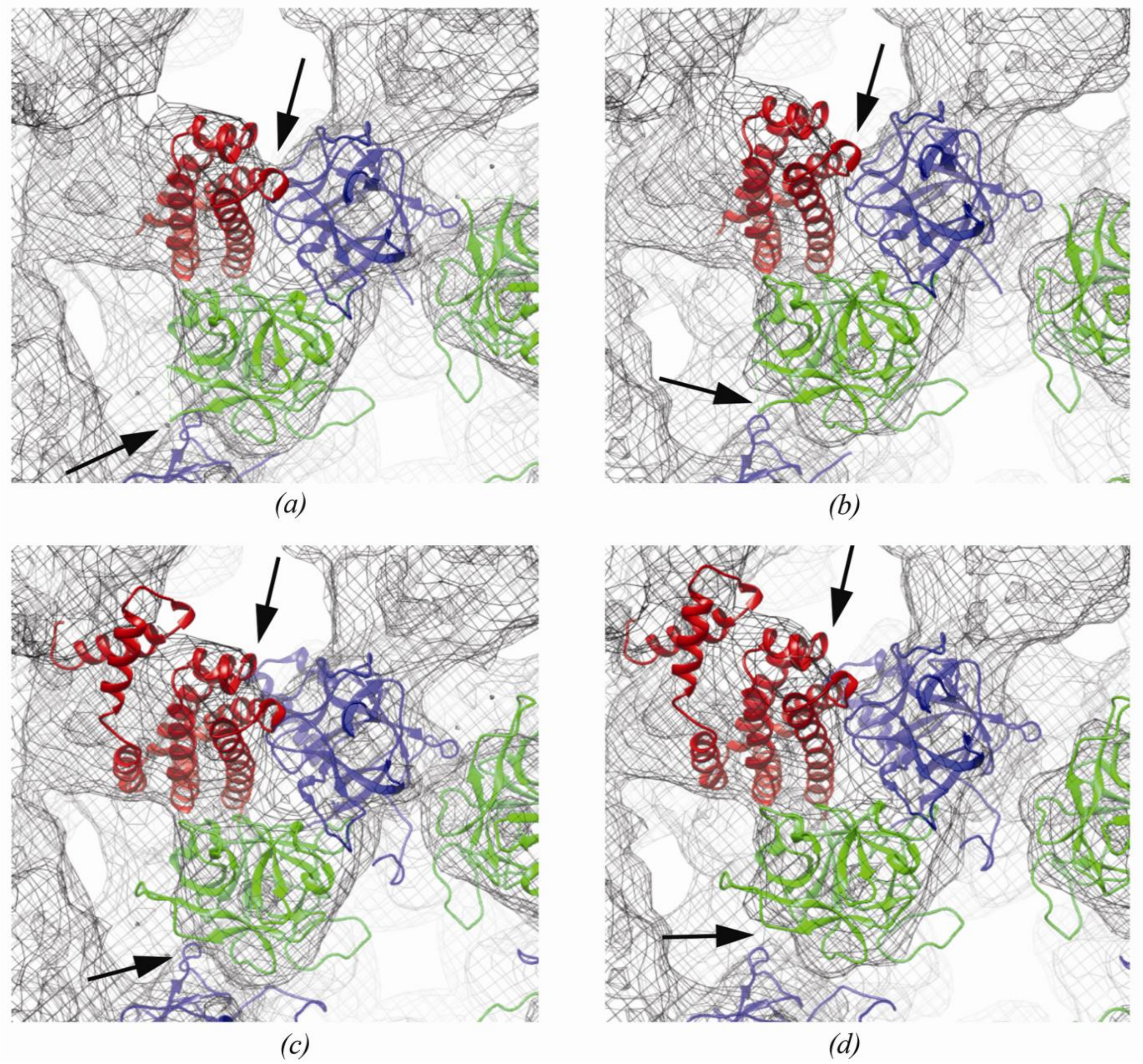

Figure S3 The hRyR2$^{1-606}$ structures docked into cryo-EM maps of the closed and open RyR1. (a, b) 4JKQ; (c, d) homology model; (a, c) closed RyR (EMD 1606); (b, d) open RyR (EMD 1607). Domains A, B, C are shown in blue, green, and red, respectively. Main differences between the closed and open conformation of RyR are denoted by arrows.

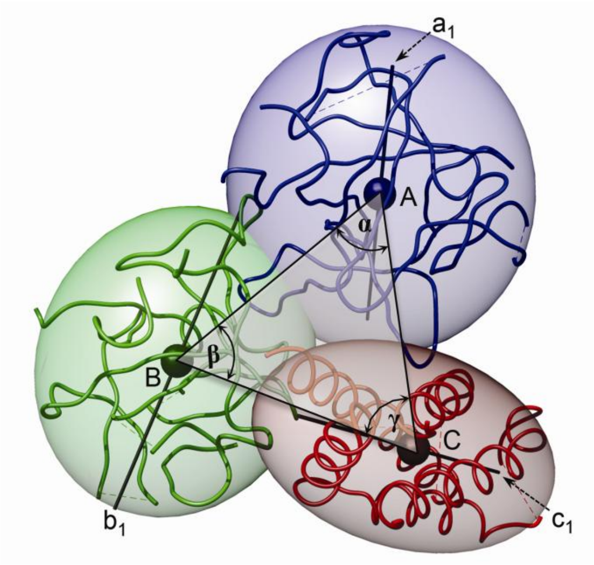

Figure S4 Representation of the A, B, C domains by inertia ellipsoids. A, B, C domains (blue, green, and red, respectively) are represented by inertia ellipsoids of the CA atoms common for all N-terminal calcium release channel structures (4JKQ, 4L4H, 2XOA, 4L4I, 3UJ0, 3UJ4, 3T8S). The centers and the longest axes (a_1, b_1, c_1) are shown as circles and lines, respectively; α, β, γ are the angles ∡BAC, ∡ABC, and ∡ACB of the triangle ABC formed by the centres of the ellipsoids.

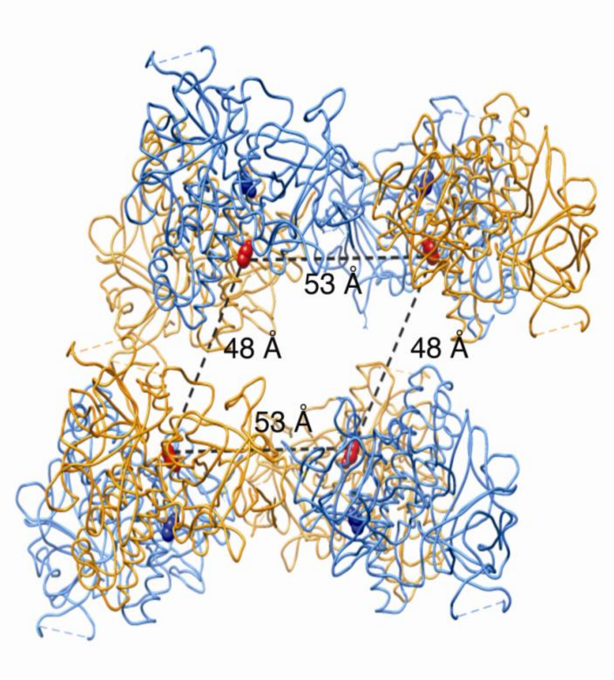

Figure S5 The cavity among the symmetry related molecules in the crystal. The molecules, in which the last visible residue (Asn544, red spheres) is facing the cavity, are shown in red, while the molecules, in which Asn544 is facing to cavities above and below the depicted region, are shown in blue.
